# The NSL Complex Regulates Housekeeping Genes in *Drosophila*

**DOI:** 10.1371/journal.pgen.1002736

**Published:** 2012-06-14

**Authors:** Kin Chung Lam, Friederike Mühlpfordt, Juan M. Vaquerizas, Sunil Jayaramaiah Raja, Herbert Holz, Nicholas M. Luscombe, Thomas Manke, Asifa Akhtar

**Affiliations:** 1 Max-Planck Institute of Immunobiology and Epigenetics, Freiburg im Breisgau, Germany; 2 Faculty of Biology, University of Freiburg, Freiburg, Germany; 3 EMBL European Bioinformatics Institute, Wellcome Trust Genome Campus, Cambridge, United Kingdom; 4 Okinawa Institute of Science and Technology, Kunigami-gun, Okinawa, Japan; Cancer Research UK Cambridge Research Institute, United Kingdom

## Abstract

MOF is the major histone H4 lysine 16-specific (H4K16) acetyltransferase in mammals and *Drosophila*. In flies, it is involved in the regulation of X-chromosomal and autosomal genes as part of the MSL and the NSL complexes, respectively. While the function of the MSL complex as a dosage compensation regulator is fairly well understood, the role of the NSL complex in gene regulation is still poorly characterized. Here we report a comprehensive ChIP–seq analysis of four NSL complex members (NSL1, NSL3, MBD-R2, and MCRS2) throughout the *Drosophila melanogaster* genome. Strikingly, the majority (85.5%) of NSL-bound genes are constitutively expressed across different cell types. We find that an increased abundance of the histone modifications H4K16ac, H3K4me2, H3K4me3, and H3K9ac in gene promoter regions is characteristic of NSL-targeted genes. Furthermore, we show that these genes have a well-defined nucleosome free region and broad transcription initiation patterns. Finally, by performing ChIP–seq analyses of RNA polymerase II (Pol II) in NSL1- and NSL3-depleted cells, we demonstrate that both NSL proteins are required for efficient recruitment of Pol II to NSL target gene promoters. The observed Pol II reduction coincides with compromised binding of TBP and TFIIB to target promoters, indicating that the NSL complex is required for optimal recruitment of the pre-initiation complex on target genes. Moreover, genes that undergo the most dramatic loss of Pol II upon NSL knockdowns tend to be enriched in DNA Replication–related Element (DRE). Taken together, our findings show that the MOF-containing NSL complex acts as a major regulator of housekeeping genes in flies by modulating initiation of Pol II transcription.

## Author Summary

Housekeeping genes are required to support basic cellular functions and are therefore expressed constitutively in all tissues. Although the homeostasis of housekeeping gene expression is vital for cell survival, most research on the transcription initiation has been focused on TATA-box-containing promoters of inducible and developmental genes, while regulatory mechanisms at the TATA-less promoters of housekeeping genes have remained poorly understood. Using genome-wide chromatin binding profiles, we find that the NSL complex, a histone acetyltransferase-containing complex, is bound to the majority of constitutively active gene promoters. We show that NSL-bound genes display specific sets of DNA motifs, well-defined nucleosome free regions, and broad transcription initiation patterns. In addition, we show that the NSL complex regulates the recruitment of the basal transcription machinery to target promoters; more specifically, we can pinpoint its role to the early steps of Pol II recruitment. Interestingly, we also see that NSL-bound genes are most susceptible to Pol II loss after depletion of NSLs when they contain the DNA Replication–related Element (DRE). Taken together, we provide a genome-wide analysis of a chromatin-modifying complex that is globally involved in the regulation of housekeeping gene expression.

## Introduction

In the past decade, our understanding of eukaryotic transcriptional regulation has changed from the notion of a “generic entity that functions by a single universal mechanism” [1] to the acknowledgement of diversity in promoter types and functions. Indeed, eukaryotic transcription relies on a complex interplay between DNA binding motifs, covalent histone modifications, higher order chromatin structures and protein-protein interactions. For example, post-translational modifications of histones such as acetylation, methylation, phosphorylation, ubiquitinylation, and sumoylation are prominent mechanisms employed to help modify chromatin structure and are considered to be a prerequisite for the recruitment of general transcription factors (GTFs) (for review see [2], [3]). Histone acetylation can impact chromatin structure in several ways: it has been shown that acetylation at different lysine residues can be specifically recognized by distinct protein domains (e.g. bromodomains) [4], [5], which in turn recruit chromatin-remodeling factors. Alternatively, acetylation itself may also disrupt interactions between nucleosomes and thus cause chromatin decompaction [6], [7]. Both mechanisms can contribute to reduced nucleosome occupancies at transcriptional start sites (TSSs), thereby providing an open chromatin environment for GTF binding [8].

Histone acetyltransferases (HATs) and histone deacetylases (HDACs) work in concert to orchestrate a fine balance of acetylation. HATs can be classified into two predominant families: the GCN5-related *N*-acetyltransferase (GNAT) family (e.g. Gcn5 and p300) [9] and the Moz-Ybf2/Sas3-Sas2-Tip60 (MYST) family (e.g. Tip60 and MOF) [10]. These enzymes often function as part of multi-protein complexes, presumably to increase substrate-specificity and to impose tight regulation of their enzymatic activity. Moreover, mounting evidence suggests that a single HAT can often associate with more than one complex [11]. Gcn5, for example, is a member of both the SAGA and ATAC complexes [12], [13] that regulate different sets of inducible genes despite sharing the same HAT [14]–[18].

Similarly MOF, a MYST-HAT specific for H4K16 acetylation, is also a member of two distinct protein complexes in *Drosophila* and mammals: the Male-Specific Lethal (MSL) and the Non-Specific Lethal (NSL) complexes [19]–[21]. In *Drosophila*, the MSL complex is targeted to the transcribed regions of X-chromosomal genes where it mediates dosage compensation. The targeting mechanism and modes of action of the MSL complex have been studied extensively (for review see [22]–[24]). In contrast, details of the NSL complex have only recently started to emerge. Our previous work revealed that the NSL complex is composed of at least seven proteins: NSL1, NSL2, NSL3, MCRS2, MBD-R2, WDS and MOF [20], [21]. We have also shown that these proteins are essential for the viability and development of *Drosophila* and that they are required for the recruitment of MOF to the promoters of active genes [21], [25]. Using a reporter assay system, Becker and colleagues demonstrated that MOF displays greater potential for transcriptional activation as part of the NSL complex, than in the MSL complex [26]. Additionally, recent reports indicate that in mammals MOF fulfills different functions in the NSL and MSL complex, respectively. It has been shown, for example, that the mammalian NSL1/MOF sub-complex appears to have broader substrate specificity than the MSL1/MOF sub-complex, as it is also able to acetylate non-histone targets [27]. Despite these observations, our understanding of NSL complex targeting and its regulatory function is still limited. Since the complex is conserved from *Drosophila* to mammals [20], unraveling its mechanism of action will be crucial for a better understanding of transcriptional regulation in higher eukaryotes and its evolutionary plasticity.

In order to elucidate the principles that direct NSL targeting, here we have performed a detailed analysis of the NSL binding sites in the genome of *Drosophila melanogaster*. We tested whether the NSL complex binds differently in distinct cell types by comparing ChIP-seq profiles obtained from the salivary glands of third instar larvae and from the Schneider (S2) cell line; our analyses reveal that the repertoire of NSL-bound genes is highly similar between different cell types. Remarkably, by comparing NSL target genes with transcriptome data from 30 distinct developmental stages of *Drosophila*, we find that the NSL complex preferentially targets genes that are constitutively expressed, also referred to as housekeeping genes. Moreover, NSL-bound genes exhibit elevated levels of H3K4me2/3, H3K9ac and H4K16ac and display a distinctive arrangement of the nucleosome free region (NFR) as well as dispersed transcription initiation patterns. Going beyond the study of NSL complex localization, we could furthermore show that the NSL complex is required for optimal recruitment of Pol II and the pre-initiation complex to its target promoters. Finally, using a quantitative model of DNA-protein interaction affinities, we find that the presence of strong DRE motifs in NSL target promoters conveys an increased sensitivity for Pol II loss in cells lacking NSL1 or NSL3. Taken together, our observations reveal a unique promoter configuration that is indicative of NSL binding and establishes the NSL complex as an important transcriptional regulator of constitutively expressed genes in *Drosophila*.

## Results/Discussion

### NSL complex targets a core set of genes independently of cell type

Genome-wide mappings of two NSL components (NSL1 and MCRS2) were previously performed in the salivary glands of third instar larvae [21]. Here, in addition, we performed chromatin immunoprecipitation followed by high-throughput sequencing (ChIP-seq) analyses of two additional proteins, NSL3 and MBD-R2, using the *Drosophila* embryonic Schneider (S2) cell line. This strategy allowed us to study similarities and differences in DNA binding patterns of the NSL-complex members in tissues of different origins. Moreover, the use of S2 cells offered the possibility to directly compare our results with the publicly available data generated by the modENCODE project that uses the same cell type.

**Figure 1:**
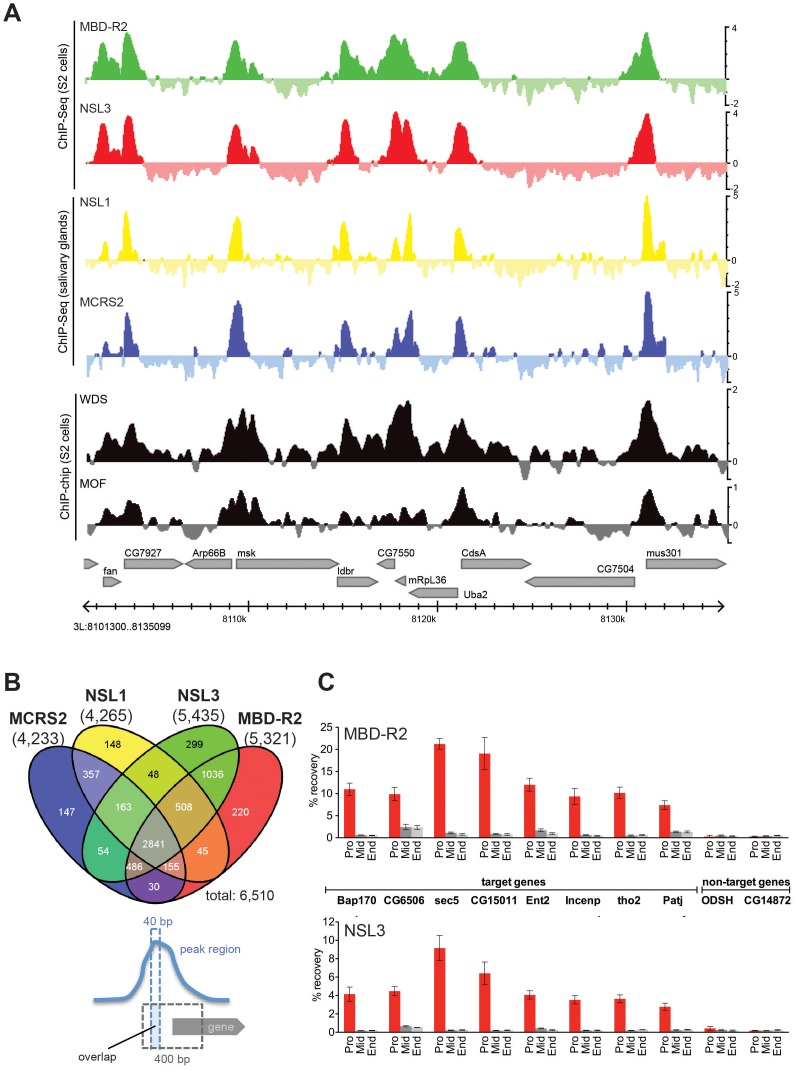
NSL proteins concomitantly bind to 5′ end of genes. (A) Genome Browser snapshot of a gene-rich region on chromosome 3 L. The log_2_FCs (ChIP/input) of the newly generated ChIP-seq data of MBD-R2 and NSL3 are compared to those of NSL1, MCRS2 [21], WDS (GEO: GSE20835) and MOF (GEO: GSE27806). (B) The Venn diagram of NSL-bound TSS regions reveals an extensive set of promoters that are concomitantly bound by all four NSL proteins. As indicated in the cartoon below the Venn diagram, a promoter was called NSL-bound if the 400 bp region surrounding the TSS (gray dashed lines) overlapped with the summit region of a peak determined by MACS and PeakSplitter (dashed blue lines). Using this definition, we identified a total of 6,510 TSSs bound by at least one NSL protein and 2,841 bound by all four. The numbers below the ChIP-ed protein names indicate the numbers of bound TSSs. (C) Chromatin immunoprecipitation followed by quantitative real-time PCR for a set of NSL target genes *(Bap170*, *CG6506*, *sec5*, *CG15011*, *Ent2*, *Incenp*, *tho2*, *Patj)* and non-target genes *(ODSH*, *CG14872)* confirm the results of the genome-wide ChIP-seq analyses: NSL proteins predominantly bind to the 5′ end of genes. Primers were designed to target the promoter (Pro), middle (Mid) and end (End) of genes; error bars represent standard deviations obtained from three independent experiments.

The four proteins displayed significant binding, ranging from 9,409 (NSL3) to 12,234 (MCRS2) genomic regions where peaks were detected (false-discovery rate <5%; statistics for individual proteins are provided in Table S1, for details on data processing see Materials and Methods). As shown in Figure S1A (dark and light blue columns), the majority of ChIP-seq peak summits localize within 800 bp of an annotated Transcription Start Site (TSS). The strongest binding signals (signified by high ChIP-seq tag counts) are observed within 200 bp of TSSs (Figure S1B, Figure 1A). We therefore focused our further analysis on NSL binding in TSS regions.

We defined a gene as NSL target when a ChIP-seq peak summit region (40 bp) was located within +/−200 bp of its TSS (see schematic representation, in Figure 1B). Using this criterion, we identified 4,233, 4,265, 5,435, and 5,321 promoters bound by NSL1, MCRS2, NSL3 and MBD-R2, respectively. Particularly in promoter-proximal regions, the binding profiles of NSL1, NSL3, MCRS2 and MBD-R2 are remarkably similar and also significantly overlap with previously published ChIP-chip profiles of WDS and MOF (Figure 1A). Despite the different developmental origins of the tissues used for the analysis of NSL1/MCRS2 and NSL3/MBD-R2, we observe that 78.7% (p-value<2.2 e-16; Fisher's exact test) of promoters with significant NSL signals are in common between the samples from S2 cells and larval salivary glands (Figure 1B). We identified a core set of 2,841 genes that are bound by all four NSL complex subunits, suggesting that the NSL proteins mostly operate as a single complex to regulate large numbers of genes in the *Drosophila* genome (Figure 1B, Figure S1C). Furthermore, ChIP followed by quantitative real time PCR (ChIP-qPCR) analysis of eight targets confirmed preferential binding of NSL proteins to the 5′-ends of genes (Figure 1C).

Given the similarity in binding between the subunits, subsequent analyses were based on the stringent core set of 2,841 genes that are bound by all four NSL proteins (thereafter called NSL-bound genes) unless otherwise indicated.

### NSL complex targets are defined by an active chromatin state

**Figure 2:**
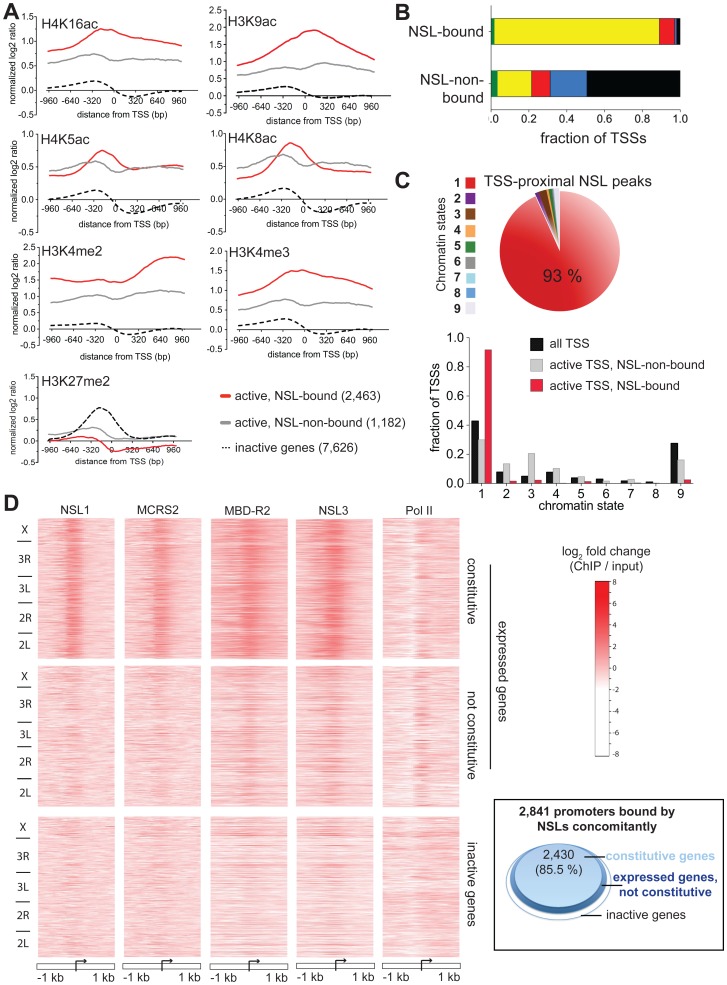
NSL proteins preferably associate with the promoters of constitutively active genes. (A) Metagene profiles of histone modifications reveal higher ratios of active chromatin marks H3K4me2/3, H4K16ac and H3K9ac for active genes bound by the NSL complex compared to active NSL-non-bound and inactive genes. On the contrary, the repressive mark H3K27me2 is not enriched on gene promoters bound by the NSL complex. Active genes were defined according to the expression data from [28] (see Materials and Methods). The expression levels of NSL-bound and NSL-non-bound active genes are similar (Figure S2A). The log_2_ ratios ( = log_2_FC (ChIP/input)) of the histone modifications were obtained from modENCODE, extracted for 200 bp bins, and normalized to H4 Chip-chip signals. (B) The chromatin color model contains [29] two states of euchromatin: “yellow” and “red”. NSL-bound TSSs are predominantly associated with “yellow”, but not “red” chromatin. NSL-non-bound genes display chromatin color ratios that resemble the pattern seen by Filion et al. for tissue-specific genes. (“Green” and “blue” correspond to classical and PcG heterochromatin, respectively, while “black” denotes regions of repressive chromatin). (C) For a different model of chromatin states devised by Kharchenko et al., similar results as in Figure 2B were obtained: The pie chart depicts that 93% of all peaks of NSL1, MCRS2, NSL3 and MBD-R2 that localize within +/−200 bp of the nearest TSS associate with regions of chromatin state 1. This is defined as the state of actively transcribed TSSs [30]. Complementary, as shown in the bar chart, NSL-bound TSSs of expressed genes are significantly enriched in chromatin state 1 and depleted of chromatin state 9 (p-values<2.2e-16; binomial test) while NSL-non-bound genes are more equally distributed between states of active TSSs (1) and elongation marks (states 2, 3, 4). (D) Heatmaps of ChIP-seq signals (log_2_FCs) demonstrate the strong enrichment of NSL binding around the TSSs of constitutively transcribed genes. In contrast to the Pol II signal that is present in both constitutive and regulatory (not constitutive) active genes, the NSL proteins are predominantly found around the TSSs of constitutively transcribed genes. As indicated on the left-hand side, genes were sorted according to their genomic location. The proteins' binding intensities can be directly compared between the different panels. The inlay (right) illustrates the findings of the heatmap with a focus on genes that are bound by all NSLs concomitantly: 85.5% of NSL-bound promoters are constitutively expressed (light blue area). Active (not constitutive) and inactive genes are represented by dark blue and white areas, respectively.

We find that 68% and 66% of actively transcribed genes in S2 cells (based on expression analysis in [28]) are bound by NSL3 and MBD-R2, respectively (p-value<2.2e-16, Fisher's exact test); similar results were obtained for NSL1 and MCRS2 from salivary glands (Table S1, [21]). To assess the relationship between gene expression, chromatin state and NSL binding, we utilized the large set of histone modification data available from the modENCODE project (see Materials and Methods for accession numbers). Surprisingly, the patterns of histone acetylation and methylation markedly differed among expressed genes depending on the presence or absence of the NSL complex. While hallmarks of transcriptionally active promoters, H3K4me2, H3K4me3, H4K16ac and H3K9ac are present regardless of NSL binding, promoters that are bound by the NSL complex show an even greater enrichment of these marks compared with active promoters that lack NSL binding (Figure 2A). These enrichments of active histone marks cannot be explained by expression level differences between the two groups (Figure S2A).

The increased acetylation of H4K16 among NSL-bound genes is in agreement with the HAT activity of MOF. However, despite a recent report by Conaway and colleagues that showed that the human NSL/MOF complex can also catalyze H4K5 and H4K8 acetylation [19], we did not observe a similar enrichment of these histone marks on NSL-bound genes. One possible explanation is that the NSL/MOF complex in *Drosophila* may have different substrate-specificity for histone residues other than H4K16 compared with humans. Alternatively, since the H4K5 and H4K8 acetylation described above was detected using an *in vitro* system, these modifications may not arise from the primary activity of MOF *in vivo*. In summary, our results indicate that the NSL-complex-bound active genes are enriched for distinct sets of histone modifications when compared with active NSL-non-bound promoters.

To gain a more comprehensive understanding of the combinations of histone modifications found at NSL-bound promoters, we studied the distribution of NSL-bound and -non-bound promoters within the five principal chromatin types (chromatin colors) defined by the location maps of 53 chromatin proteins [29]. Within this model, the chromatin states “yellow” and “red” correspond to active genes, but differ in the combination of histone marks and chromatin binding proteins. Unexpectedly, we found a very significant enrichment of NSL-bound TSSs for the “yellow” chromatin state that is associated specifically with MRG15 and H3K36me3 (87.3% versus 18.2% for NSL-non-bound; p-value<2.2e-16; Fisher's exact test; see Figure 2B), but no comparable enrichment for the “red” chromatin state that is marked by chromatin proteins, such as Brahma, SU(VAR)2–10 and MED31 (9.7% of NSL-bound TSSs versus 8% of NSL-non-bound TSSs for “red”; Figure 2B). Our findings suggest the NSL complex as an additional, previously unknown marker of “yellow” chromatin while genes within “red” chromatin regions are expected to undergo NSL-independent transcriptional regulation.

A similar dominance for one specific state of active chromatin was observed when we repeated the analysis for the 9-chromatin-state model developed by Kharchenko and co-workers [30] (Figure 2C, Figure S2B), supporting the notion of the NSL complex as a regulator of a particular set of actively transcribed genes.

### The NSL complex predominantly targets housekeeping genes

The results of the chromatin state analyses and the fact that most NSL binding appears to occur independently of the cell-type, prompted us to examine whether the complex displayed any association with housekeeping genes. To address this question, we defined a set of genes that are constitutively expressed throughout 30 distinct developmental stages of *Drosophila* [31] as our list of housekeeping genes (see Materials and Methods). We then generated heatmaps for Pol II and NSL binding centered on the TSSs of annotated genes [32] that were classified into three classes: constitutively expressed genes (see above), active genes but not expressed throughout all developmental stages of the fly [28], [31] and inactive genes. As shown in Figure 2D, the Pol II signal shows the anticipated enrichment downstream of the TSSs of active genes regardless of constitutive or tissue-specific expression. In striking contrast, the NSL binding profiles show a very prominent, almost exclusive enrichment around the TSSs of constitutively expressed genes but not among those active genes that show tissue-specific regulation. Accordingly, 91.6% of NSL3-bound genes, 89.6% of MBD-R2-bound genes and 85.5% of TSSs bound by all four NSLs concomitantly belong to the group of housekeeping genes (Table S1, inlay in Figure 2D). Conversely, out of 5,534 constitutively expressed genes, 4,950 (89.4%; p-value<2.2e-16, Fisher's exact test) were bound by at least one NSL protein (Figure S2C, S2D). This number is likely to be an underestimation as some of the constitutively expressed genes, which are classified as NSL-non-bound according to our strict criteria, also show detectable NSL protein signals (Figure S2E). Taken together, we concluded that the NSL complex preferentially binds to constitutively expressed genes.

### NSL-bound promoters have dispersed transcription initiation patterns and distinct nucleosome organization

**Figure 3:**
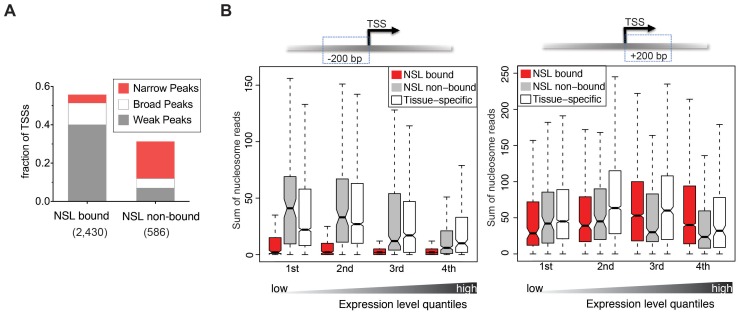
NSL-bound genes display a specific nucleosome organization at their TSS. (A) The TSSs of constitutively active genes, either NSL-bound or –non-bound, were analyzed regarding their reported transcription initiation patterns [35]. NSL-bound TSSs mostly belong to genes with weak and broad transcription initiation peaks (40% and 11.4%) whereas NSL-non-bound TSSs mainly belong to genes with narrow transcription initiation peaks (19.3%). (B) Boxplots of the sum of overlapping nucleosome reads in the regions 200 bp upstream and 200 bp downstream of the TSSs of constitutively expressed NSL-bound genes (red), constitutively expressed NSL-non-bound genes (gray), and tissue-specific genes (white). Genes were stratified based on their gene expression quartile (see Materials and Methods) which demonstrates that the depletion of nucleosomes immediately upstream of the TSS that we observed for NSL-bound housekeeping genes (left side) is independent of expression levels (p-values for −200 bp region <2.2e-16; Wilcoxon test).

In addition to expression-based definitions of housekeeping genes, we wanted to test further correlations of NSL binding with characteristics of constitutively expressed genes. Earlier studies have revealed two basic types of *Drosophila* promoters based on the pattern of the transcriptional initiation: broad and peaked [33]–[36]. While broad promoters preferably belong to housekeeping genes, peaked promoters are associated with tissue-specific expression. Based on data from [35], we found that NSL-bound TSSs are predominantly associated with dispersed transcription initiation patterns (Figure 3A).

We next wanted to investigate whether the NSL-characteristic initiation patterns and histone modifications enrichments also connected to specific structural features of the chromatin. Genome-wide analyses of nucleosome-positioning have demonstrated that transcriptionally active genes display a distinct organization, consisting of a precisely located +1 nucleosome around 135 bp downstream of the TSS, a −1 nucleosome that is directly upstream of the TSS and a nucleosome free region (NFR) between them. Additionally, it has been shown that the nucleosome organization can vary quite dramatically depending on the promoter sequences and transcription initiation patterns [37], [38].

To assess whether NSL-bound promoters display a specialized nucleosome arrangement, we integrated a recently published map of nucleosome positions in S2 cells [38]. First, we examined the nucleosome occupancy for 4,950 constitutively expressed genes bound by at least one NSL protein, 717 constitutively expressed NSL-non-bound genes, and a set of 6,138 genes with tissue-specific expression (Figure S3; see Materials and Methods). For NSL-bound constitutively expressed genes we observe a well-defined nucleosome organization: Nucleosomes located within 200 bp upstream of the TSSs are strongly depleted while nucleosomes along the gene body are well positioned. In contrast, constitutively expressed genes not bound by the NSL complex (as well as tissue-specific genes) display a very different organization that is characterized by a less pronounced NFR and rather fuzzy positioning of the nucleosomes (Figure S3). This is in line with previous studies where more defined nucleosome positioning was associated with specific promoter sequences [37] and broad transcription initiation patterns [38].

The distinct nucleosome occupancies for NSL-bound genes prompted us to test if the observed difference in nucleosome positioning was related to gene expression levels. The analysis of the promoter proximal regions of NSL-bound, NSL-non-bound and tissue-specific genes revealed that the diminished nucleosome occupancy upstream of the TSS is, in fact, independent of the expression levels (Figure 3B).

### NSL1 and NSL3 are required for efficient recruitment of Pol II on target promoters

Since the NSL complex predominately targets gene promoters, we next addressed whether its presence is important for the recruitment of RNA Polymerase II (Pol II). For this purpose, we first depleted NSL1, NSL3 and MBD-R2 in S2 cells by dsRNA-mediated depletion. The efficiency of the knockdown was assessed by Western blot analyses of nuclear or cytoplasmic extracts from the relevant cells (Figure S4A). Consistent with previous observations [21], NSL1 depletion had the most severe effect on the stability of NSL2, NSL3 and MCRS2. In contrast, MOF levels remained unaffected or at most showed a modest decrease upon MBD-R2 depletion. Interestingly, in comparison to the severe reduction of overall protein levels for NSL complex members, levels of Pol II, TBP and TFIIB showed almost no or only modest effects upon NSL1, NSL3 and MBD-R2 depletion.

We also assessed the quality of NSL1, NSL3 and MBD-R2 depletion by performing chromatin immunoprecipitation with NSL1, NSL3 and MBD-R2 antibodies in NSL-depleted versus control cells (dsRNA against GFP). Consistent with the Western blot analyses, the ChIP experiments revealed severe depletion of NSL1, NSL3 and MBD-R2 from target promoters (Figure S4B).

**Figure 4:**
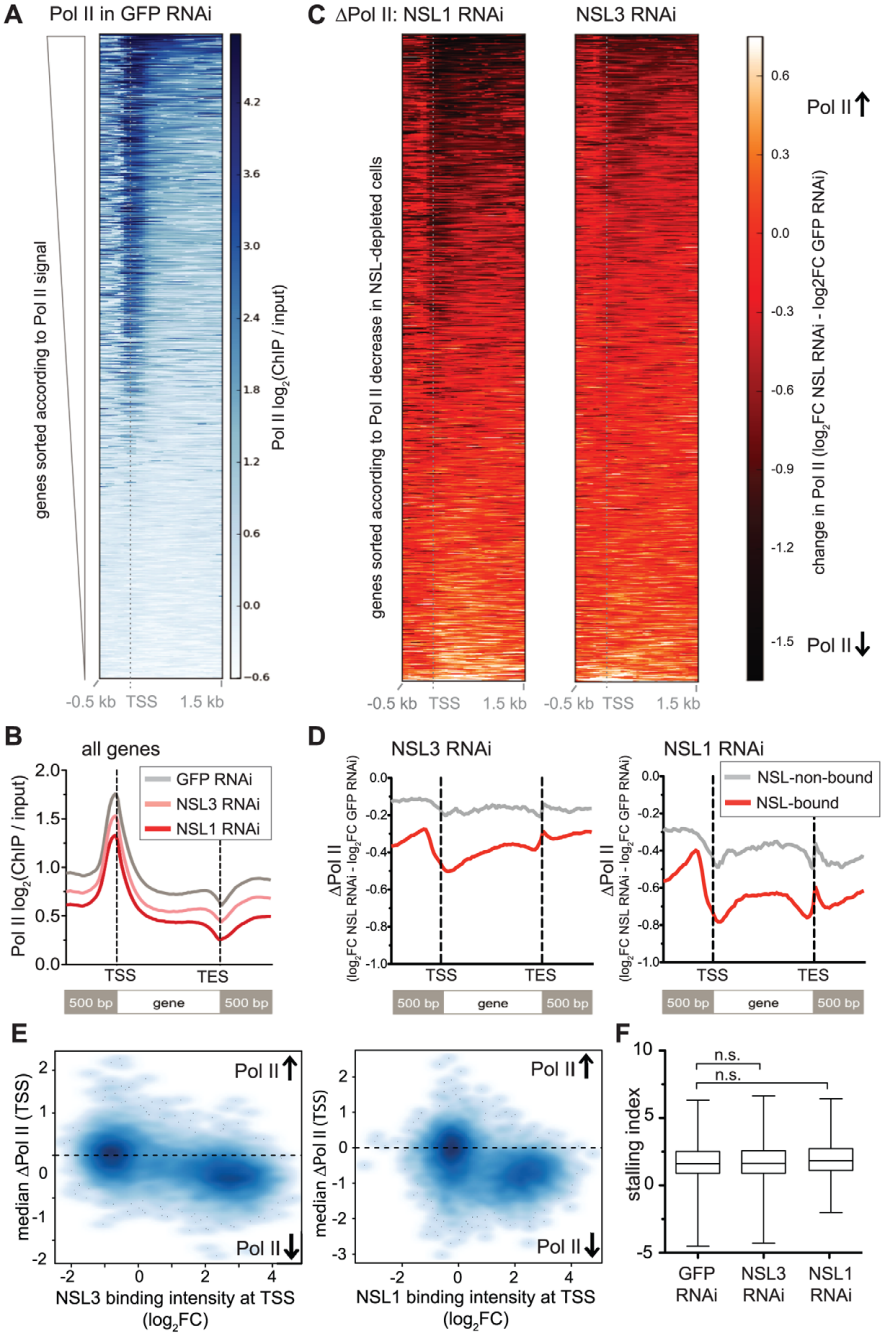
NSL depletion leads to Pol II loss on target genes of the NSL complex. (A) The heatmap displays input-normalized Pol II binding signals for 5′ ends of *D. melanogaster* genes as captured by ChIP-seq of Rbp3 in S2 cells that had been treated with dsRNA against GFP. Genes were sorted according to the signal strength: Genes with high Pol II binding on promoters as well as along the gene bodies are found in the upper part of the heatmap. They are followed by genes with Pol II binding primarily at the promoter and genes lacking detectable Pol II signals. (B) Metagene profiles of the genome-wide signals of Pol II shows a marked decrease of Pol II binding for cells lacking NSL1 or NSL3 compared to control cells. (C) Here, the change of Pol II binding upon knockdown of NSL1 and NSL3 (ΔPol II) was visualized. The ΔPol II signal is calculated as the difference of normalized Pol II ChIP-seq signal (log_2_FC) in NSL-depleted cells and control cells. Genes are ranked according to the change of Pol II in NSL knockdown; genes with greatest Pol II loss are found at the top of the heatmap. Severe reduction of Pol II after NSL depletion is seen around the TSSs and along gene bodies (dark red to black color), but there are also numerous genes that are slightly or not affected (bright red color). (D) Average ΔPol II values were plotted for active genes, separated into NSL-bound and –non-bound ones. The general decrease of Pol II upon NSL knockdown was observed again. In addition, it now becomes more evident that the magnitude of Pol II loss is markedly higher in NSL-bound genes compared to NSL-non-bound genes. (E) To study the association between the loss of Pol II (i.e. negative ΔPol II values) and NSL binding in an unbiased manner, median ΔPol II values at promoters were plotted against the median binding intensities of NSL1 and NSL3 from wild type samples. Genes were filtered for non-overlapping genes and those with significant Pol II binding in the control sample; the promoter region was defined as a 400 bp region centered around the TSS. The scatter plots confirm that genes with substantial NSL signals show markedly lower ΔPol II values than genes without NSL binding (left hand side of the plot). The difference of ΔPol II between NSL-bound and NSL-non-bound genes is statistically highly significant as determined by Wilcoxon rank sum test (p-value<2.2e-16). The observation that the majority of the genes with high NSL binding display a negative ΔPol II value (Pol II loss), suggests the NSL complex as a transcriptional activator whose binding to genes has functional consequences. (F) Stalling indexes for all genes with significant Pol II binding in control and NSL-depleted cells were calculated. Stalling indexes are derived from the ratio of Pol II at the promoter versus Pol II along the gene body (see Materials and Methods); high stalling indexes indicate Pol II accumulation at the promoter and diminished release into transcriptional elongation. No statistically significant difference between the stalling indexes of genes in the three different conditions was observed (median stalling indexes are 1.611 for GFP-RNAi treated cells, 1.649 in NSL3-RNAi treated cells and 1.848 in NSL1-RNAi, p-value>0.1, Wilcoxon rank sum test; n.s. = not significant).

Following these quality criteria, we proceeded with genome-wide ChIP-seq analyses of Pol II in NSL1- and NSL3- depleted cells (Figure S5). As shown in Figure 4A, we obtained well-defined enrichments of Pol II binding at both the promoters and along the gene bodies of active genes in the GFP knockdown sample. The accumulation of Pol II at promoters is consistent with previous reports and indicative of widespread Pol II stalling [38]. When examining the global effects of the NSL knockdowns on Pol II levels, we observed a marked decrease in Pol II levels around transcription start sites (Figure 4B, 4C), particularly on genes that we had previously identified as bound by the NSL complex (Figure 4D). The loss of Pol II was even more pronounced in cells lacking NSL1 compared to those lacking NSL3. This effect could have been the consequence of different knockdown efficiencies of dsRNA against NSL1 and NSL3. Additionally, Western blot analyses of the individual NSL proteins revealed different effects of NSL1 and NSL3 depletion on NSL complex stability (see above and Figure S4A). Since protein levels of the other NSL complex members were either mildly affected or unaffected following the knockdown of NSL3, the remaining NSL complex members might have been able to partially continue transcriptional support in the absence of NSL3. This could explain the less severe effects of NSL3 depletion on Pol II binding compared to NSL1 depletion.

Regardless of the difference in the magnitude of Pol II reduction, both knockdowns showed greater effects on NSL-bound genes compared to NSL-non-bound active genes, suggesting that the NSL complex directly promotes the recruitment of Pol II to promoters of its target genes (Figure 4D, 4E). To assess whether the decrease of Pol II signal along the gene body could be attributed to elevated stalling of Pol II at the promoter, we calculated stalling indexes as described in [39] (see Materials and Methods). We could not detect a significant increase in the median stalling index (1.611 in GFP knockdown compared to 1.848 in NSL1 knockdown and 1.649 in NSL3 knockdown samples, p-value>0.1 as determined by Wilcoxon rank sum test, see Figure 4F). The unaffected stalling indexes suggest that NSL depletion does not interfere with the transition of Pol II from initiation to elongation. Taken together, these results strongly suggest that the NSL complex is required for efficient recruitment of Pol II at its target promoters.

### NSL1, NSL3, and MBD-R2 are required for efficient recruitment of general transcription factors

Pol II recruitment to promoters is a multi-step process requiring the assembly of a functional pre-initiation complex (PIC). In the current model, the TFIID complex (containing TBP) first binds to core promoter regions where it is stabilized by TFIIA and TFIIB. TFIIF and Pol II are subsequently recruited to the core promoter by TFIIB [40], [41]. Since we had established a general role of the NSL complex for Pol II recruitment, we now sought to identify the specific initiation step that was affected by NSL depletion.

**Figure 5:**
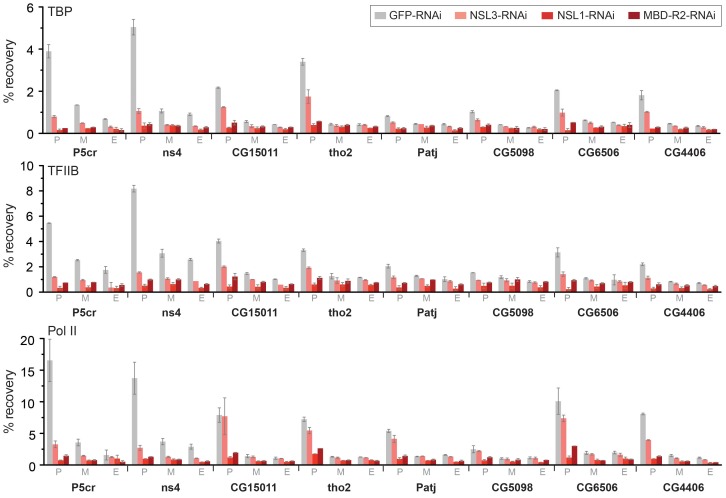
The NSL complex is important for optimal recruitment of the pre-initiation complex. ChIP was performed with antibodies against TBP, TFIIB and Pol II (Rpb3) in NSL1, NSL3 and MBD-R2 depleted S2 cells as well as in GFP knockdown control cells. The quantitative qPCR was performed on six autosomal genes (*P5cr*, *ns4*, *CG15011*, *tho2*, *Patj*, *CG5098*) as well as 2 X-linked genes (*CG6506* and *CG4406*). Primers were positioned at the promoter (P), middle (M) and end (E) of the indicated genes. Percentage recovery is determined as the amount of immunoprecipitated DNA relative to input DNA. Error bars represent the standard deviation between independent experiments.

Our next step was to perform ChIP-qPCR studies of individual NSL target genes following the knockdown of NSL1, NSL3 or MBD-R2. The results revealed that both TBP and TFIIB binding was decreased at promoters, indicating an interruption in the early stage of PIC assembly (Figure 5). In contrast to NSL complex members, TBP and TFIIB protein levels did not show a severe reduction upon NSL1 and NSL3 knockdown (Figure S4A). Consistent with previous observations [21], we did not detect a major difference in H4K16ac levels upon NSL1, NSL3 or MBD-R2 knockdown, possibly due to remaining MOF protein, or slow turnover of H4K16ac or the nucleosomes (Figure S6). Taken together, these data suggests that NSL1, NSL3 and MBD-R2 are required for efficient recruitment of TBP/TFIIB to target promoters presumably for efficient PIC formation.

### DRE and motif 1 are associated with Pol II loss caused by NSL depletion

Distinct classes of gene expression patterns, e.g. constitutive or tissue-specific gene expression, are associated with particular promoter DNA motifs. Yet, how the presence or absence of a DNA motif is translated into biological functions often remains elusive. Since the NSL complex preferentially binds housekeeping genes, we wanted to investigate putative underlying DNA motifs and associate them with the effects of NSL depletion on Pol II recruitment.

**Figure 6:**
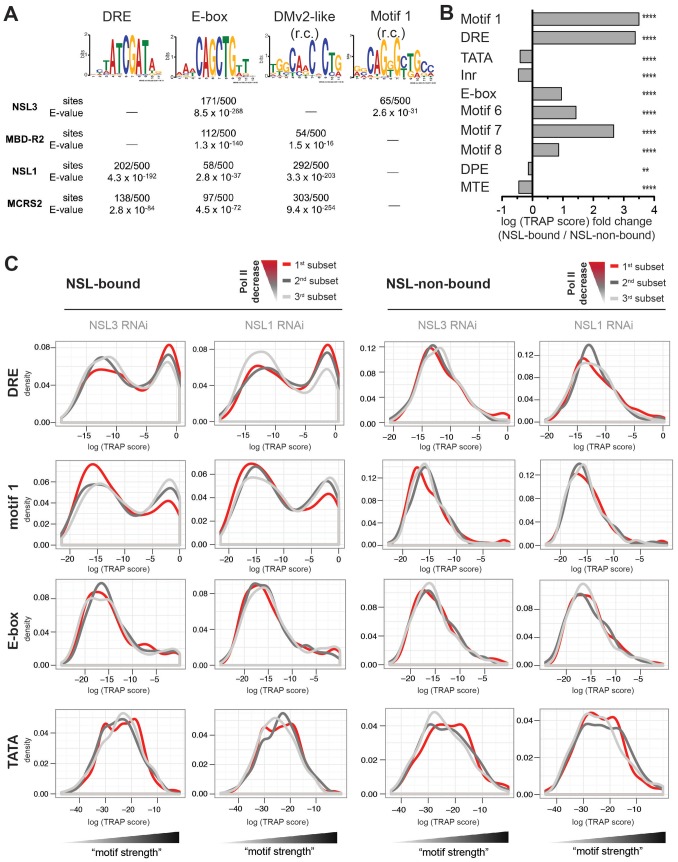
NSL target regions are enriched for housekeeping gene motifs, but only DRE and motif 1 are directly related to Pol II loss upon NSL depletion. (A) Individual *de novo* motif analysis led to the discovery of four non-repetitive DNA motifs that are located within NSL-complex binding sites (r.c. = reverse complement). The analysis was carried out by MEME [62], [64] for 100 bp regions around the peak summits. As computational restrictions of MEME allowed only a limited number of base pairs to be analyzed at a time, the results of the 500 highest peak regions are shown here (for additional peak regions see Figure S7). (B) Motif enrichments were calculated with TRAP [44], [63] using the motif matrices for the 10 known core promoter motifs identified by [43]. In our study, the TRAP score can be seen as a measure for the affinity of transcription factors to bind to the DNA regions of interest. We compared the TRAP scores for NSL-bound and –non-bound promoter regions (TSS +/−200 bp) and found Ohler motifs 1, 6, 7, 8 as well as DRE and E-box significantly and selectively enriched in NSL-target regions while TATA box, Inr, DPE and MTE are depleted. The bar plot depicts the fold change between the median TRAP scores of NSL-bound versus –non-bound regions; individual frequency distributions of the motifs' TRAP scores can be seen in Figure S8 (for constitutitve gene promoters). P-values for the comparison of NSL-bound versus –non-bound promoters were calculated with two-sided Wilcoxon rank sum test, **** = P<0.0001, *** = P<0.001, ** = P<0.01, * = P<0.5. (C) To determine the significance of the Ohler motifs for the function of the NSL complex, genes were divided into three classes according to the magnitude of Pol II loss. The 1^st^ subset (red line) corresponds to genes with the most severe Pol II reduction upon NSL knockdown while the 3^rd^ subset (light gray line) contains least affected genes. Density distributions of TRAP scores were then plotted for NSL-bound and –non-bound genes for each Ohler motif individually. For DRE and motif 1 there is a clear distinction between the differently affected NSL-bound genes: NSL targets that lose Pol II binding most dramatically after NSL knockdown (red line) are clearly enriched for high DRE TRAP scores. In contrast, motif 1 shows an inverse pattern compared to DRE: NSL-bound genes with mild Pol II loss (light gray line) tend to contain strong motif 1 sites. This trend is not observed in NSL-non-bound genes. Other motifs such as E-box and TATA box also did not show significant association (also see Figure S9).

We first assessed which motifs were enriched in NSL target regions: The unbiased *de novo* motif finder MEME repeatedly identified four known core promoter elements within NSL peak regions: the E-box motif (CAGCTG), DRE (WATCGATW), the reverse complement of a motif resembling DMv2 (TGGYAACR [42]) and motif 1 (YGGTCCACTR [43]; Figure 6A, Figure S7). Applying a quantitative model of transcription factor binding affinities (TRAP) to the 10 well-known *Drosophila* core promoter motifs [43], [44], we detect a strong enrichment for DRE and E-box as well as Motifs 1, 6, 7, 8 in NSL-bound promoters compared with non-bound ones (p-values<0.0001, Wilcoxon rank sum test; Figure 6B, Figure S8). This is in complete concordance with previous genome-wide studies that suggested a preference of housekeeping genes for these motifs [34], [35].

We have shown that the NSL complex is crucial for Pol II recruitment to housekeeping genes. However, Figure 4E reveals variability in the extent of Pol II loss among genes with high NSL binding signals. This is in line with the observation published by Becker and colleagues [45]. One possible explanation could be that different core promoter motifs underlie the variable responses of NSL-bound genes to NSL loss. We thus assessed whether the motif strengths is associated with the impact of NSL depletion on Pol II recruitment. For this purpose, we stratified NSL-bound and –non-bound genes into three subsets according to the magnitude of Pol II loss on promoters and plotted the corresponding distribution densities for each motif's strength (Figure 6C, Figure S9).

Based on the equally strong enrichment of motif 1 and DRE (see Figure 6B) one might have expected a similar importance of these motifs for the function of the NSL complex. Interestingly, when we integrated the genome-wide Pol II binding data, we observed that DRE and motif 1 are associated with Pol II loss upon knockdown of NSL complex members in opposing manners: For the DRE motif we see a positive correlation between the levels of Pol II loss and the abundance of genes with high DRE TRAP scores. Motif 1, on the other hand, is mostly associated with genes that are least sensitive to Pol II loss after NSL depletion (light gray line in Figure 6C). For NSL-non-bound genes, neither DRE nor motif 1 show any enrichment in relation to Pol II loss. Enrichment of E-box and other core promoter motifs (except motif 7, Figure S9) do not exhibit a correlation with the sensitivity to NSL complex depletions.

In conclusion, our analysis demonstrates that NSL-bound promoters are enriched for core promoter motifs DRE, E-box and motif 1, 6, 7, 8 and depleted for TATA, Inr, DPE and MTE sequences. Even more importantly, the presence of DRE motifs is positively associated with the degree of responsiveness of NSL target genes to NSL complex depletion.

### Summary

In this study, we have revealed that the majority of the NSL-complex-bound targets are housekeeping genes in *Drosophila*. While chromatin-modifying complexes that regulate tissue-specific genes, such as SAGA, polycomb and trithorax complexes, have been studied extensively, global regulators of housekeeping genes are poorly understood. To our knowledge, the NSL complex is the first identified major regulator of housekeeping genes which is consistent with a recently published study from Becker and colleagues [45].

**Figure 7:**
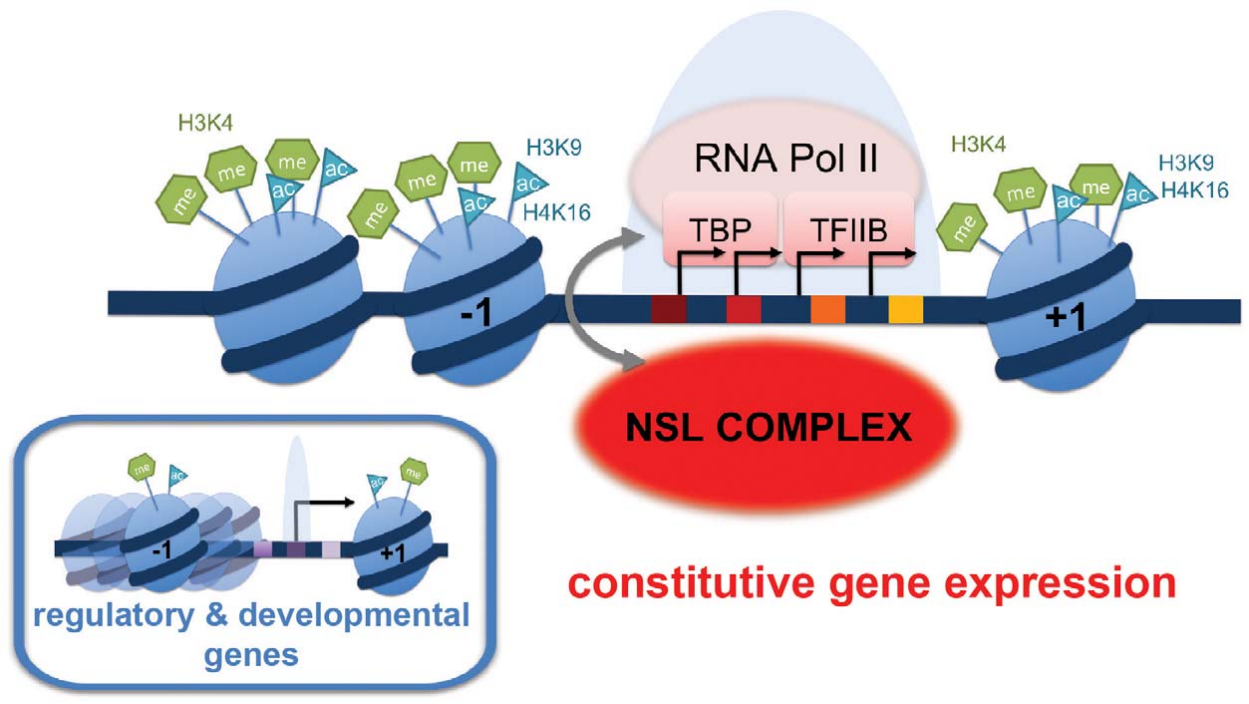
Summary model: NSL-dependent Pol II recruitment to promoters of housekeeping genes. The majority of the NSL-bound targets are constitutively expressed or “housekeeping” genes. These genes are characterized by prominent enrichment of particular histone modifications (H4K16ac, H3K9ac, H3K4me2, H3K4me3) as well as specific core promoter elements (such as DRE, E-box and motif 1; indicated by colored squares). In contrast, tissue-specific or developmentally regulated genes (small inlay) usually contain the TATA-box as the most prominent core promoter element. We propose that the NSL complex acts as a regulator of constitutively expressed genes by facilitating stable recruitment of the pre-initiation complex (PIC) members such as Pol II, TBP and TFIIB on target genes. NSL complex may therefore serve as an important link between specific promoter architecture and PIC assembly.

The promoters of NSL target genes exhibit prominent enrichment of certain histone modifications (H4K16ac, H3K9ac, H3K4me2, H3K4me3) as well as specific core promoter elements (such as DRE, E-box and motif 1). Furthermore, these genes display distinct nucleosome occupancy and dispersed promoter configuration characterized by multiple transcription start sites. The correlation between these promoter characteristics (well-defined chromatin marks, TATA-less DNA sequences and broad initiation patterns) was previously identified for housekeeping genes in mammals and flies [36], but how these promoter features are translated into gene transcription had remained elusive. We now conclusively demonstrate that the NSL complex modulates transcription at the level of transcription initiation by facilitating pre-initiation complex loading onto promoters. Therefore, we propose that the NSL complex is a key *trans*-acting factor that bridges the promoter architecture, defined by the DNA sequence, histone marks and higher chromatin structures with transcription regulation of constitutive genes in *Drosophila* (Figure 7).

Excitingly, the enrichment of DNA motifs on NSL target gene promoters in combination with the genome-wide Pol II binding data has established functional links between the motifs enriched on housekeeping genes and the NSL-dependent Pol II binding to promoters. The abundance of DRE motifs, for example, was found to be positively associated with the magnitude of Pol II loss upon NSL knockdowns. The DRE binding factor (DREF) interacts tightly with TRF2 to modulate the transcription of DRE-containing promoters in a TATA-box-independent fashion [46]. It is tempting to speculate that the NSL complex might also cooperate with the TRF2 complex to facilitate transcription in a specific manner, rendering DRE-containing promoters more sensitive to NSL depletions. As the NSL-bound promoters are associated with a large variety of transcription factors, it will be of great interest to study whether the NSL complex communicates with different transcription regulators, perhaps making use of distinct mechanisms.

In contrast to DRE, motif 1 showed an opposing effect on Pol II recruitment to NSL-complex-bound genes as the presence of strong motif 1 sequences was associated with decreased Pol II loss upon NSL depletion. The mechanistic reasons for this remain unclear. However, one can envisage several possible scenarios. It is possible that motif 1 may recruit another transcription factor, which can also function to recruit the transcription machinery. Alternatively, the turnover of the transcription machinery might be slower on promoters containing strong motif 1 sequences. There is precedent for the transcription machinery having various turnover rates on different promoters. For example, in yeast, it has been shown that TBP turnover is faster on TATA-containing than on TATA-less promoters [47]. It is therefore possible that certain levels of the initiation complexes may still be maintained on motif-1-containing promoters, even though the recruitment of the transcription machinery will be compromised in the absence of NSL complex. Further work is required to understand the importance of sequence determinants for NSL complex recruitment and our analysis sets the grounds for targeted experiments in the future.

Taking MOF-mediated H4K16 acetylation into consideration, a putative role of the NSL complex might be to coordinate the opening of promoter architecture by histone acetylation and the assembly of PIC. Coupling of histone acetylation and PIC formation has been described before. For example, TAF1, a component of TFIID, is a histone aceyltransferase [48]. The SAGA complex, which contains Gcn5 and can acetylate H3K9, is reported to interact with TBP and other PIC components to regulate tissue-specific genes [49], [50] and the recruitment of P300 to the promoter and H3 acetylation have been shown to proceed binding of TFIID in a coordinated manner [51]. H4K16ac is also well-known for its role in transcription regulation of the male X chromosome, yet how H4K16 acetylation and PIC assembly are coordinated remains elusive. Interestingly, absence of the NSL complex does not severely abolish H4K16ac from target genes. Since the turnover of H4K16ac on target promoter is unknown, it remains possible that H4K16ac could remain for some time at the promoter after the NSL complex is depleted. Further studies will be crucial in unraveling the functional relevance of H4K16 acetylation and NSL complex function on housekeeping genes.

## Materials and Methods

### Chromatin immunoprecipitation (ChIP) and real-time PCR

Chromatin immunoprecipitation was carried out on S2 cells as previously described [21]. Fixed chromatin was sheared into 200 bp fragments and probed with antibodies against *Drosophila* TBP, TFIIB, Pol II, H4K16ac (sc8662, Santa Cruz), H4 (ab7311, Abcam), NSL1, MCRS2, NSL3 and MBD-R2 [21].

Real-time PCR validation was performed with SYBR-Green PCR master mix (Applied Biosystems) and an ABI7500 real-time PCR thermocycler (Applied Biosystems, Inc.). Recovery was determined as the amount of immunoprecipitated DNA relative to input DNA.

### Deep sequencing of ChIP samples

Deep sequencing of NSL3 and MBD-R2 ChIP and input samples was carried out with the Illumina Genome Analyzer II, Pol II ChIP (from GFP-RNAi, NSL1-RNAi, NSL3-RNAi) and respective input samples were deep-sequenced with an Illumina HiSeq2000 machine according to manufacturer's instructions.

### Mapping

The sequence reads from our earlier study of NSL binding in salivary glands [21] and the newly generated data from S2 cells were aligned to the *D. melanogaster* genome (dm3) using bowtie [52] with identical parameters. We allowed up to two mismatches and reported only the best alignments which could be aligned uniquely. We obtained 28,335,659 and 27,328,733 uniquely mapped reads for NSL3 and MBD-R2 respectively (input: 24,271,994 reads). The re-mapping of the NSL1 and MCRS2 data resulted in 7,622,096 and 9,405,874 unique reads (input: 6,168,473 reads).

From the samples sequenced with HiSeq 2000, we obtained between 120 to 135 million reads for Pol II ChIPs from S2 cells with knockdowns of NSL1, NSL3, and GFP and 50 to 60 million reads for the corresponding input samples. The correlations between the biological replicates of Pol II ChIP-seq reads from NSL1 and NSL3 knockdowns were excellent (Figure S5).

### Peak calling

We used MACS Version 1.4.0rc2 on bed-files of mapped reads from the ChIP-seq experiments of NSL1, MCRS2, NSL3, MBD-R2, and an input control. We employed standard parameters for *D. melanogaster* (including model-building) and a p-value cut off of 10^−5^ [53]. We invoked PeakSplitter [54] as part of the MACS routine to obtain subpeak coordinates. For downstream analyses we used the subpeaks of peaks with a false discovery rate ≤5%. Unless noted otherwise, peak summit regions were henceforth defined as the 40 bp region surrounding a summit identified by PeakSplitter.

Previous ChIP-seq analyses of Pol II have revealed that there are at least two types of Pol II signals: a sharp peak around the TSS of genes that can be either expressed or stalled, and an additional wide-spread region of moderate enrichment over the body of genes which is associated with transcription elongation, pausing and termination. The composite nature of the Pol II signal is not captured optimally by MACS, therefore we normalized the read counts (per 25 bp bins) of the Pol II ChIP-seqs with the BioConductor package DESeq [55], calculated the log_2_ fold changes (log_2_FC) between library-size-normalized input and ChIP samples and applied a 400 bp sliding window to account for the fragment size obtained after sonication. To determine regions of significant Pol II enrichment we modeled the distribution of log_2_FC values based on negative log_2_FCs that are assumed to correspond to experimental noise. We calculated the threshold log_2_FC values for significant Pol II binding within the three different conditions at an FDR-value cut-off of 0.05 (method described in more detail in [56]). The threshold log_2_FCs were 0.64, 0.84 and 1.39 for Pol II signals from GFP-RNAi, NSL3-RNAi, and NSL1-RNAi, respectively.

### Lists of genes and associated NSL peaks

The basis of our gene-focused analyses was the list of annotated genes from FlyBase (version 5.30). Genes that are active in S2 cells were obtained from [28]. Data from [31] was used for a list of constitutively active genes: 5,534 genes expressed above a significance threshold (set by [31]) in all 30 developmental stages of *D. melanogaster* were considered constitutively expressed (housekeeping genes). To identify genes that were active in S2 cells, but not constitutively expressed, the gene identifiers of the different lists were adapted with the help of the FlyBase ID converter tool and subsequently subtracted from each other.

Unless indicated otherwise, a TSS was defined NSL-bound when the 400 bp region surrounding the TSS overlapped with an NSL peak summit region. The scripts, BEDTool commands and Galaxy workflows used for these overlaps and analyses are available upon request [57], [58].

### Calculation of Pol II stalling indexes

For the calculation of the stalling indexes we first applied stringent filters to the genes that were taken into account: we included only non-overlapping genes greater than 1,300 bp and with median Pol II signals above the threshold (see above) at the promoter region. Promoter regions were defined as TSS +/−200 bp, for the gene body regions we excluded 500 bp after the TSS and 500 bp before the transcription end site (TES) to avoid confounding effects of transcription initiation and termination. Based on previous reports by Muse et al. [39], the stalling index (SI) itself was calculated as follows: SI = log_2_(*r* (TSS)/*r* (gene body)) where *r* is the sum of Pol II ChIP-seq read counts that were adjusted by the input sample and normalized to the region's length.

### Calculation of ΔPol II

To assess the change of Pol II upon NSL depletion in comparison to the GFP-RNAi control sample, ΔPol II was calculated as follows: ΔPol II = log_2_FC (NSL-RNAi)−log_2_FC (GFP-RNAi).

### Graphical representations

We visualized the binding profiles of the NSL complex proteins with our locally installed GBrowser (Version 2.15), uploading normalized log_2_FCs and wiggle files from modEncode.

For the summary plots of the histone marks (Figure 2A), we extracted the log_2_FCs from publicly available ChIP-chip data: The 2,000 bp TSS regions were split into 100 bins and the average log_2_FCs were calculated for each bin and normalized to the corresponding H4 signals.

For the heatmaps shown in Figure 2D we divided the annotated genes (FlyBase version 5.30) into active and inactive in S2 cells [28]. Active genes were further classified as constitutively and not-constitutively transcribed according to [31] (see above). For each gene, we extracted the normalized log_2_FCs (ChIP/input) from our ChIP-seq data (NSL1, MCRS2, NSL3, MBD-R2) and published ChIP-chip data of Pol II [32] in 50 bp bins for 1,000 bp up- and downstream of the TSSs. The heatmaps were generated with R using the same scale for every individual image and maintaining the order of the underlying TSS lists to enable direct comparisons between the different binding profiles on the same genes. Mitochondrial genes were excluded.

For the heatmaps of Pol II and ΔPol II (Figure 4A, 4C) we used all *D. melanogater* genes except mitochondrial genes. The log_2_FC of Pol II from the GFP-RNAi sample and ΔPol II (see above) for NSL3-RNAi and NSL1-RNAi were extracted in 50 bp bins for the regions 500 bp up- and 1,500 bp downstream of each gene's TSS. Genes were sorted according to the cumulative signal within the displayed region as indicated in the respective figures and legends.

For the metagene profiles of Pol II and ΔPol II signals as shown in Figure 4B and 4D, gene bodies of non-overlapping, size-filtered genes were scaled to the same length; log_2_FCs (ChIP/input) were extracted accordingly. Venn diagrams were generated with Venny [59].

### Nucleosome occupancy analysis

We measured nucleosome occupancy for constitutively expressed NSL-bound genes, constitutively expressed NSL-non-bound genes and tissue-specific genes in a 200 bp area surrounding their annotated TSSs (4,971, 717 and 6,138 genes respectively). Nucleosome maps for S2 cells were obtained from GEO (accession number: GSE22119 [38]). NSL-bound genes for this analysis were defined as those bound by any of the NSL1, NSL3, MCRS2 or MBD-R2 subunits. Constitutive genes were defined as in [31] (see above). Tissue-specific genes were selected as in [60] on the basis of the ‘gene scores’ derived from Affymetrix tiling arrays for 25 different cell lines and 30 developmental stages (modENCODE accession number: modENCODE_3305). In order to avoid any bias in nucleosome organization due to differences in gene expression levels, genes were stratified in quartiles according to their expression value (ArrayExpress: E-MEXP-150 [61]). Finally, for each gene, nucleosome occupancy was calculated as the sum of overlapping reads with a 200 bp area up- and downstream its TSS.

Nucleosome metaprofiles were calculated using the average sum of overlapping reads with 25 bp bins spanning the area 500 bp up- and 1000 bp downstream of the TSS of each gene.

### MEME

We sorted the peaks identified by MACS and PeakSplitter according to their summits' tag counts and extracted the DNA sequences for a 100 bp region centered around them. The peak summits were analyzed by MEME [62] in subsequent analyses of 500 sequences each with the following parameters: revcomp, nmotifs = 3, minw = 6, maxw = 12, minsites = 10.

### Motif enrichment: TRAP analysis

In addition to the *de novo* motif analysis by MEME, we studied sequence properties of NSL-targets using 10 motif matrices from the supplementary material of [43]. As transcription factors can bind to DNA with a range of affinities, we employed a biophysical model (TRAP [44], [63]) that predicts the binding affinity for a motif in a given sequence fragment. We refer to the logarithm of this number as the TRAP score that indicates the strength of the putative protein-DNA interaction for each Ohler motif within a region of interest. The TRAP score enables us to quantitatively assess the corresponding binding affinities, i.e. we do not rely on the binary classification of motif presence or absence. Instead we are able to compare the “protein binding capacity” of different regions of interest.

We applied the TRAP model to NSL binding sites and promoter regions, which we defined as +/−200 bp around the TSS. To assess the localization of the binding signals, more precisely, the TRAP score was calculated for sliding windows of 40 bp over this region. The average TRAP scores for each window were then compared between specific sets of promoters regions (NSL-targets and -non-targets).

To assess the relation between ΔPol II and the TRAP score (Figure 6C, Figure S9), we focused on the promoter regions of non-overlapping genes with median Pol II signal (log_2_FC) above the threshold value in GFP-RNAi and ΔPol II below 0 (i.e., loss of Pol II upon NSL knockdown). Tertiles based on ΔPol II were determined with the quantile function of R. We used a 100 bp window around the TSS for TRAP score calculation for all motifs except TATA (40 to 20 bp upstream of the TSS), Inr (TSS +/−20 bp), and DPE (20 to 40 bp downstream of the TSS). Density plots were generated with the R package ggplot2.

### Chromatin state associations

We downloaded the bed-files with the genomic coordinates of the 9-state-chromatin model of [30] for S2 cells and the chromatin color model of [29] from modENCODE and identified the number of peak summits (2 bp) or TSSs intersecting with the different states. We also divided the peak summits into three groups according to their overlap with annotated TSSs: proximal (within +/−200 bp), peripheral (between +/−201–800 bp) and distal subpeaks (farther away than 800 bp).

### Data from public repositories

For the analysis of histone marks and non-histone chromosome proteins, we downloaded the wiggle-files of ChIP-chip experiments on S2 cells from modEncode/Gene Expression Omnibus.

H3K4me3-S2: GSE20787

H3K4me2-S2: GSE23470

H4K16ac-S2: GSE20799

H3K27me2-TJ.S2: GSE27790

H3K9ac-S2: GSE20790

H4K5ac-S2: GSE20800

H3K18ac-S2. GSE20775

MOF_Q4145.S2: GSE27806

WDS_Q2691.S2: GSE 20835

H4: repset.4620571

Pol II: GSM463297

Nucleosome maps: GSE22119

S2 gene expression data for nucleosome occupancy: E-MEXP-1505 [61]

### Primers used for qPCR

L = forward primer, R = reverse primer.

CG6506-pro-L: GCCGATGTTTACCGACAATC

CG6506-pro-R: CATGGTTGGTTATCGGGACT

CG6506-Mid-L: ATCCGTGCCTAATGATACCG

CG6506-Mid-R: ACGGTTGGTGTGAACCAAAT

CG6506-end-L: ACAGTCAGCTCCCAGCAGAT

CG6506-end-R: AAAGTGGCGTGAAAGTTGCT

Sec5-pro-L: GCTGCTCAGCAAGGAGACTT

Sec5-Pro-R: CGGACGAGCATAAAAAGAGC

Sec5-mid-L: GAACTCCCATTGGCGATAAA

Sec5-mid-R: AAATGTCTGGCGAAATGTCC

Sec5-end-L: ATCAACGGCTTCATCTTTCG

Sec5-end-R: GCGTTTTCTTCCATTTTCCA

ODSH-Pro-L: CCCATTTTTCCCACTGACTG

ODSH-Pro-R: GGCGCGTACAAATGAAAAAT

ODSH-Mid-L: AAGATCCGCTAAGCGATGAA

ODSH-Mid-R: GCCAGGAGTTGAAGTTGGTC

ODSH-End-L: AGGCTCTCGTGGGGTAAAAT

ODSH-End-R: GAGCTCACCGATTTGTTTCC

CG15011-pro-L: CAGCCCTGGTATTCGATGTT

CG15011-pro-R: CTCATCTTGGATCGGATCGT

CG15011-Mid-L: CCTGCCACAAGGAACACTTT

CG15011-Mid-R: AGCTGCAACAAGCACAAATG

CG15011-end-L: ACACGGTGTTCTTCCAGTCC

CG15011-end-R: CGCTAAGGAACGTCGAAATC

CG14872_Pro_L: AATCGAGACATTCAGGCACTC

CG14872_Pro_R: TTCCCACACTGAAAAATCCA

CG14872_Mid_L: AAGAGCTTGAACAGCGGAAC

CG14872_Mid_R: GATACGCAAACCGGCATC

CG14872_End_L: TCACGCTCTAAAACCCCAGA

CG14872_End_R: CAGTACGGCATGGGCAAC

Patj_Pro_L: GAGTGCATAGGAGAGGGTAAACA

Patj_Pro_R: GTGGCGTTGGCACACTTT

Patj_Mid_L:CGTCGGTCACCACAATGA

Patj_Mid_R: TTATCCGCCAAGGGTACAAC

Patj_End_L: ACGCGGTTGCTAACTAATGG

Patj_End_R: ACTTCTGGCATCGTTTCTGAC

tho2_Pro_L: CCTCGGATCAGGTGGTACA

tho2_Pro_R: GTCACACTGGCGGAACTAACT

tho2_Mid_L: GGCCACATCCGTGTTTATGT

tho2_Mid_R: GCCAAGACACACTCGTCCA

tho2_End_L: GCTTCACAATGCACGGAAC

tho2_End_R: GAGGAGCGGCAGTACATCA

Ent2_Pro_L: CGTAACGGCACCCCTCAA

Ent2_Pro_R: ACCGCACCGCACTACAAG

Ent2_Mid_L: CCGCCATCCTAGTGCTGCT

Ent2_Mid_R: GCTGCTCCGGCTAATGGT

Ent2_End_L: TCTCGTATCTGGGACCATTTT

Ent2_End_R: TCCCGGAACTGGTATTGAG

Bap170_Pro_L: CCTGCTCGTGAATGCAACT

Bap170_Pro_R: GTGGCGTGAATGGGAAAC

Bap170_Mid_L: ACCCCCAGCATTGTTCCT

Bap170_Mid_R: CTTTCCTCAGACGCCACTTC

Bap170_End_L: ATGAACCGACACACGACTGA

Bap170_End_R: GCCGTAGCCGAGTAGGTGA

Incenp_Pro_L: GTTCTTTCCCTTACCATTTTCC

Incenp_Pro_R: GTTCCCGCCACTACCATCT

Incenp_Mid_L: GAGGACGAGTCGGTGGAG

Incenp_Mid_R: TTGAAAAGCTCATGTGTACGG

Incenp_End_L: GCCACGTAAGGGGAGAGG

Incenp_End_R: GTTCGGGAATATCTGCTTTAGG

ns4_Pro_L: GAGATGCCAACTTGTAGGTGATT

ns4_Pro_R: AAATACATGCAGAGACAGGAGGT

ns4_Mid_L: GCAAGGTGGTCAGCGTTAGT

ns4_Mid_R: GACTAGACCGGGACAATCACA

ns4_End_L: GACAGCGAGGATGAAGACGA

ns4_End_R: CAGCAGAGCAAACACGTTCC

CG5098-Pro-L: GGTCTTGTTTATGGGCGAAA

CG5098-Pro-R: GAGGGAAAGGCGACCTAATC

CG5098-Mid-L: GATGAGCCTCCCAAAAATCA

CG5098-Mid-R: GGCTACTTTGGCTGCTATGC

CG5098-End-L: GGGCATTTCGTAATCCAAGA

CG5098-End-R: TTTGGGGAAGGGAACCTAAC

p5cr_Pro_L: CACACCAAAGCTCAGAGGAGT

p5cr_Pro_R: CCGATTGCATGGGCGTAG

p5cr_Mid_L: GCGAGGGCTGCACTGTTT

p5cr_Mid_R: TGGACTCGGGCACCTGTT

p5cr_End_L: ATGTAATCCCCCGGAACA

p5cr_End_R: GCAAGAAGGATCGGGAATAA

CG4406-pro-R: TATCGACGGTCACACTGCTC

CG4406-mid-L: CCTGGAACTTGAGGAATCCA

CG4406-mid-R: GGCAGCAATGTGCTCATCTA

CG4406-end-L: AGCTCGGAAGGAAACTGTGA

CG4406-end-R: GTGACCAAAAAGCCCTTCAA

### RNAi in S2 cells

RNAi of S2 cells was performed as described previously [21]. All knockdown cells were transfected with 10 µg dsRNA against NSL1, NSL3, MBD-R2 or GFP using Lipofectamine RNAiMAX (Invitrogen) and were harvested after 6 days. EGFP control RNAi experiments were performed in parallel.

### RNAi sequences used to generate dsRNA for the following genes

NSL1:

*T7-NSL1 sense*: 5′- TTA ATA CGA CTC ACT ATA GGG AGA ATG GCC CCA GCG CTC ACA-3′

*T7-NSL1 antisense*: 5′- TTA ATA CGA CTC ACT ATA GGG AGA TGA ACT TGT GGC CAC TGC C-3′

NSL3:

*T7-NSL3 sense*: 5′- TTA ATA CGA CTC ACT ATA GGG AGA TCC TTG GCG ACT ACC TCA TC-3′

*T7-NSL3 antisense*: 5′- TTA ATA CGA CTC ACT ATA GGG AGA GTA CCA TTT CGG CCC CTA GTG-3′

MBD-R2:

*T7-MBD-R2 sense*: 5′- TTA ATA CGA CTC ACT ATA GGG AGA CGC TGG CCA CGT TTA TTA AG-3′

*T7-MBD-R2 antisense*: 5′- TTA ATA CGA CTC ACT ATA GGG AGA TTG AAG AGA AAA AGC TTG TAC GG-3′

EGFP:

*T7-EGFP sense*: 5′-TA ATA CGA CTC ACT ATA GGG AGG ATG GTG AGC AAG G

*T7-EGFP antisense*: 5′-TA ATA CGA CTC ACT ATA GGG AGG ATC GCG CTT CTC G

### Accession numbers

All ChIP seq data is available in the ArrayExpress database (http://www.ebi.ac.uk/arrayexpress/) with the accession numbers listed below.

NSL1 and MCRS2 ChIP-Seq from salivary glands: E-MTAB-214

NSL3 and MBD-R2 ChIP-Seq from S2 cells: E-MTAB-1085

Pol II ChIP-Seq from S2 cells (GFP-RNAi, NSL1-RNAi, NSL3-RNAi): E-MTAB-1084

## Supporting Information

Figure S1 General characteristics of NSL binding profiles. (A) ChIP-Seq peaks obtained from NSL profiles were classified according to their distance from the nearest annotated TSS. The bar chart shows that the majority of NSL binding events is closely associated with annotated TSSs: 68.7% of NSL3 peaks, 67% of MBD-R2 peaks, 81.5% of NSL1 peaks, and 76.1% of MCRS2 peaks localize within 800 bp up- or downstream of the nearest TSS. The schematic diagram below the bar chart visualizes our definitions: proximal peaks localize within +/−200 bp (dark blue), peripheral peaks between 201–800 bp (light blue) and distal peaks are farther away than 800 bp from a TSS (white). (B) The strongest signals of NSL binding are observed within 200 bp of annotated TSSs. This is shown by the box plot of tag counts of peak summits classified as TSS-proximal, -peripheral, or –distal (whiskers = 2.5–97.5 percentiles). (C) The lack of complete overlap of NSL target genes is mainly due to stringent criteria for defining target genes. In Figure 1B, 1,036 genes were shown as “bound by NSL3 and MBD-R2 only” and 357 genes as “bound by NSL1 and MCRS2 only”. We therefore addressed whether these two groups could constitute gene sets that are specific for S2 cells or salivary glands. For this purpose, input-normalized ChIP-seq signals for the promoters for each group of genes were extracted, including those that are bound by all or neither NSL proteins. The box plot shows that the signal of NSL1 and MCRS2 is still significantly higher in those genes that were labeled as “bound by NSL3 and MBD-R2 only” than for those that were defined as NSL-non-bound (p-value<2.2e-16, Wilcoxon test). The same holds true for NSL3 and MBD-R2. Therefore, differences in gene sets are very likely not due to tissue-specific binding, rather to the choice of a very stringent cut-off for the binary decision “bound” or “not-bound”. For details about our definition of NSL target genes, see Materials and Methods and Figure 1B.
(PDF)

Figure S2 Assessing the overlaps of NSL signals on gene promoters. (A) Median expression levels between expressed genes that are bound by all four NSLs concomitantly do not differ significantly from expressed genes devoid of NSL binding as shown by the box plot (whisker = 2.5–97.5 percentiles). The expression scores were taken from [28]. (B) The NSL complex preferentially binds to regions of open and actively transcribed chromatin (state 1, [30]) as peak summits intersected with the regions reported by [30] are dramatically enriched for state 1 (regardless of their localization). (C) Overview of TSS-associated NSL binding: 19.25% of annotated TSSs are bound by NSL1, MCRS2, NSL3, and MBD-R2 concomitantly. When looking at the subsets of active and housekeeping genes, the numbers increase to 37.1% (active) and 43.9% (constitutive) that are bound by all four NSLs across different cell types and experiments. To confirm the findings that were based on our own definition of housekeeping genes (see Materials and Methods), we also tested a previously published set of broadly and restrictedly expressed genes [65]. (D) The Venn diagram shows the individual overlaps of the gene promoters bound by the single NSL proteins. The core intersect (2,430) corresponds to the gray bar of “constitutive genes” in Figure S2C, while the total number of 4,950 represents the number of constitutive TSSs bound by at least one NSL. (E) Constitutive genes classified as NSL-non-bound according to our criteria described in Materials and Methods (see Figure 1 for visualization) show slightly, but significantly elevated levels of NSL binding compared to non-constitutively expressed genes. This verifies the preference of the NSL complex for housekeeping genes and suggests that some constitutive genes classified as NSL-non-bound were missed due to the cut-off we used for all four samples. The boxplot shows the median log_2_FCs (ChIP/input) for the 400 bp regions centered around TSSs. The medians were calculated for each gene based on the ChIP-seq tags of all four analyzed NSL proteins.
(PDF)

Figure S3 NSL-bound and NSL-non-bound housekeeping genes display different nucleosome organizations. Nucleosome occupancy metaprofiles for NSL-bound (red), constitutively expressed NSL-non-bound (gray) and tissue-specific (black) genes. Metaprofiles were calculated for each group as the sum of nucleosome reads overlapping 25 bp bins spanning the −500/+1000 bp region centered at the TSS of each gene. The non-shaded white area corresponds to the −200/+200 bp region used for the analysis in Figure 3B.
(PDF)

Figure S4 Depletion of different NSL proteins have distinct effects on the stability of the remaining NSL complex members but not for Pol II machinery components. (A) Western blot analyses of cytoplasmic (C) and nuclear (N) extracts from S2 cells that had been treated with dsRNA against GFP, MBD-R2, NSL1, and NSL3. Depletion of NSL1 greatly affects the stability of other NSL complex proteins namely: NSL2, NSL3, MCRS2, MBD-R2 and WDS. Depletion of NSL3 or MBD-R2 has milder effects on the levels of other NSL proteins. MOF protein levels appear affected upon MBD-R2 depletion but not in NSL1 or NSL3 knockdowns. In contrast, TBP, TFIIB and Pol II are only modestly affected in either knockdown especially when taking into consideration the loading control Nuclear RNA export factor 1 (NXF1). (B) To check whether the dsRNA treatment against NSL3, NSL1, and MBD-R2 efficiently reduced NSL binding to its target regions, ChIP was performed with antibodies against NSL1, NSL3 and MBD-R2 in the respective knockdowns in S2 cells. GFP-RNAi was used as a control. “P”, “M”, “E” represent promoter, middle and end of gene, respectively. Error bars represent the standard deviation of three independent experiments.
(PDF)

Figure S5 Correlation of biological duplicates for the ChIP-seq of Pol II in knockdowns of NSL1 and NSL3. Correlation plots between the two Pol II ChIP-seq libraries generated from duplicate knockdown experiments for (a) NSL3 and (b) NSL1. Reads were mapped to the genome with bowtie. The read counts plotted here were extracted for 25 bp bins along the entire *D. melanogaster* genome. The Spearman correlations for the biological replicates are excellent (0.96 for NSL3-RNAi samples, 0.97 for NSL1-RNAi samples).
(PDF)

Figure S6 Chromatin immunoprecipitation of H4K16ac in NSL1, NSL3 and MBDR2 depleted cells. ChIP-qPCR was performed using antibodies against H4K16ac and H4 in NSL1, NSL3 or MBD-R2 depleted cells. The H4K16ac signal is normalized against H4 signal from the same region. Consistent with our previous results, H4K16ac is very modestly reduced upon depletion of NSL complex members. The quantitative qPCR was performed on 5 autosomal genes (*P5cr*, *ns4*, *CG15011*, *tho2*, *Patj*, *CG5098*) as well as 2 X-linked genes (*CG6506* and *CG4406*). Primers were positioned at the promoter of the indicated genes. Error bars represent the standard deviation of three independent experiments.
(PDF)

Figure S7 *De novo* motif identification in NSL binding regions. (A) Motifs identified by MEME in peak summit regions of NSL3, MBD-R2, NSL1, and MCRS2. Results of MEME analyses of peaks ranked 501–1,000 (r.c. = reverse complement) confirm the motifs identified in the 500 highest peaks as shown in Figure 6A. (B) Results of MEME analyses of 500 peak summits that were not selected solely according to their height, but also on the basis of their association with constitutively expressed genes. The motifs and their occurrences recapitulate the results from the analysis of the highest intensity peaks (Figure 6A), reinforcing the preference of NSL targeting to genomic regions containing the motifs shown above.
(PDF)

Figure S8 Comparison of motif enrichments for NSL-bound and –non-bound constitutively active TSSs. (A) The bar chart displays the fold change of the core promoter affinities for the sequences of NSL-bound promoters (concomitant binding of NSL1, MCRS2, MBD-R2, NSL3) compared to NSL-non-bound promoters (not bound by any of the NSL proteins). The bar chart shows that even when motif enrichments are calculated within the subset of constitutively active genes, NSL-bound promoters are enriched for motif 1, DRE, E-box, motif 6, 7 and 8 whereas the depletion of TATA box, Inr motif, DPE and MTE becomes less evident. P-values were calculated with two-sided Wilcoxon rank sum test, **** = P<0.0001, *** = P<0.001, ** = P<0.01, * = P<0.5, not significant (n.s.) = P>0.5. The fold change was calculated as log(median(TRAP score of NSL-bound promoters)/median(TRAP score of NSL-non-bound promoters)). (B) Individual TRAP score [44], [63] histograms for the 10 core promoter motifs [43] that underlie the bar chart of S6A. The histograms show the distributions of the motif affinities for NSL-bound and –non-bound promoters of housekeeping genes. The visible shifts towards higher or lower TRAP scores in NSL-bound or –non-bound genes, respectively, represent the fold changes seen in the bar chart.
(PDF)

Figure S9 TRAP score densities of NSL-bound and –non-bound genes. We selected non-overlapping genes that showed significant Pol II binding in control samples and reduced Pol II levels in NSL knockdown conditions and sorted them into three groups according to the magnitude of Pol II loss. The 1^st^ subset (red line) contains genes with the strongest reduction of Pol II in promoter regions; the 3^rd^ subset (gray line) correspondingly contains genes with smallest Pol II loss. TRAP score was calculated as a measure of protein binding affinity towards the known promoter motifs identified by Ohler et al. [43]. Of the motifs shown here, only motif 7 displays a moderate association between the motif's strength and Pol II loss upon NSL depletion (for remaining motifs see Figure 6C).
(PDF)

Table S1 Overview of the numbers of NSL peaks overlapping with annotated TSSs. The numbers of peaks (regions of significant NSL binding signals) determined by MACS [53] are comparable between the different proteins. However, the increased sequencing depth of the NSL3 and MBD-R2 ChIP-seq experiments led the detection of expanded regions of NSL binding signals that is reflected by more widespread peak. The PeakSplitter algorithm [54] divides peaks identified by MACS at sites of local maxima.
(PDF)

## References

[1] Juven-Gershon T, Kadonaga JT (2010). Regulation of gene expression via the core promoter and the basal transcriptional machinery.. Dev Biol.

[2] Kouzarides T (2007). Chromatin modifications and their function.. Cell.

[3] Yang XJ, Seto E (2007). HATs and HDACs: from structure, function and regulation to novel strategies for therapy and prevention.. Oncogene.

[4] Jacobson RH, Ladurner AG, King DS, Tjian R (2000). Structure and function of a human TAFII250 double bromodomain module.. Science.

[5] Ruthenburg AJ, Li H, Milne TA, Dewell S, McGinty RK (2011). Recognition of a mononucleosomal histone modification pattern by BPTF via multivalent interactions.. Cell.

[6] Luger K, Mader AW, Richmond RK, Sargent DF, Richmond TJ (1997). Crystal structure of the nucleosome core particle at 2.8 A resolution.. Nature.

[7] Shogren-Knaak M, Ishii H, Sun JM, Pazin MJ, Davie JR (2006). Histone H4-K16 acetylation controls chromatin structure and protein interactions.. Science.

[8] Shahbazian MD, Grunstein M (2007). Functions of site-specific histone acetylation and deacetylation.. Annu Rev Biochem.

[9] Vetting MW, LP SdC, Yu M, Hegde SS, Magnet S (2005). Structure and functions of the GNAT superfamily of acetyltransferases.. Arch Biochem Biophys.

[10] Utley RT, Cote J (2003). The MYST family of histone acetyltransferases.. Curr Top Microbiol Immunol.

[11] Lee KK, Workman JL (2007). Histone acetyltransferase complexes: one size doesn't fit all.. Nat Rev Mol Cell Biol.

[12] Grant PA, Schieltz D, Pray-Grant MG, Steger DJ, Reese JC (1998). A subset of TAF(II)s are integral components of the SAGA complex required for nucleosome acetylation and transcriptional stimulation.. Cell.

[13] Suganuma T, Gutierrez JL, Li B, Florens L, Swanson SK (2008). ATAC is a double histone acetyltransferase complex that stimulates nucleosome sliding.. Nat Struct Mol Biol.

[14] Huisinga KL, Pugh BF (2004). A genome-wide housekeeping role for TFIID and a highly regulated stress-related role for SAGA in Saccharomyces cerevisiae.. Mol Cell.

[15] Lebedeva LA, Nabirochkina EN, Kurshakova MM, Robert F, Krasnov AN (2005). Occupancy of the Drosophila hsp70 promoter by a subset of basal transcription factors diminishes upon transcriptional activation.. Proc Natl Acad Sci U S A.

[16] Krebs AR, Demmers J, Karmodiya K, Chang NC, Chang AC (2010). ATAC and Mediator coactivators form a stable complex and regulate a set of non-coding RNA genes.. EMBO Rep.

[17] Nagy Z, Riss A, Fujiyama S, Krebs A, Orpinell M (2010). The metazoan ATAC and SAGA coactivator HAT complexes regulate different sets of inducible target genes.. Cell Mol Life Sci.

[18] Suganuma T, Mushegian A, Swanson SK, Abmayr SM, Florens L (2010). The ATAC acetyltransferase complex coordinates MAP kinases to regulate JNK target genes.. Cell.

[19] Cai Y, Jin J, Swanson SK, Cole MD, Choi SH (2010). Subunit composition and substrate specificity of a MOF-containing histone acetyltransferase distinct from the male-specific lethal (MSL) complex.. J Biol Chem.

[20] Mendjan S, Taipale M, Kind J, Holz H, Gebhardt P (2006). Nuclear pore components are involved in the transcriptional regulation of dosage compensation in Drosophila.. Mol Cell.

[21] Raja SJ, Charapitsa I, Conrad T, Vaquerizas JM, Gebhardt P (2010). The nonspecific lethal complex is a transcriptional regulator in Drosophila.. Mol Cell.

[22] Straub T, Becker PB (2007). Dosage compensation: the beginning and end of generalization.. Nat Rev Genet.

[23] Hallacli E, Akhtar A (2009). X chromosomal regulation in flies: when less is more.. Chromosome Res.

[24] Conrad T, Akhtar A (2011). Dosage compensation in Drosophila melanogaster: epigenetic fine-tuning of chromosome-wide transcription.. Nat Rev Genet.

[25] Andersen DS, Raja SJ, Colombani J, Shaw RL, Langton PF (2010). Drosophila MCRS2 associates with RNA polymerase II complexes to regulate transcription.. Mol Cell Biol.

[26] Prestel M, Feller C, Straub T, Mitlohner H, Becker PB (2010). The activation potential of MOF is constrained for dosage compensation.. Mol Cell.

[27] Li X, Wu L, Corsa CA, Kunkel S, Dou Y (2009). Two mammalian MOF complexes regulate transcription activation by distinct mechanisms.. Mol Cell.

[28] Cherbas L, Willingham A, Zhang D, Yang L, Zou Y (2011). The transcriptional diversity of 25 Drosophila cell lines.. Genome Res.

[29] Filion GJ, van Bemmel JG, Braunschweig U, Talhout W, Kind J (2010). Systematic protein location mapping reveals five principal chromatin types in Drosophila cells.. Cell.

[30] Kharchenko PV, Alekseyenko AA, Schwartz YB, Minoda A, Riddle NC (2011). Comprehensive analysis of the chromatin landscape in Drosophila melanogaster.. Nature.

[31] Graveley BR, Brooks AN, Carlson JW, Duff MO, Landolin JM (2011). The developmental transcriptome of Drosophila melanogaster.. Nature.

[32] Nechaev S, Fargo DC, dos Santos G, Liu L, Gao Y (2010). Global analysis of short RNAs reveals widespread promoter-proximal stalling and arrest of Pol II in Drosophila.. Science.

[33] Rach EA, Yuan HY, Majoros WH, Tomancak P, Ohler U (2009). Motif composition, conservation and condition-specificity of single and alternative transcription start sites in the Drosophila genome.. Genome Biol.

[34] Hoskins RA, Landolin JM, Brown JB, Sandler JE, Takahashi H (2011). Genome-wide analysis of promoter architecture in Drosophila melanogaster.. Genome Res.

[35] Ni T, Corcoran DL, Rach EA, Song S, Spana EP (2010). A paired-end sequencing strategy to map the complex landscape of transcription initiation.. Nat Methods.

[36] Rach EA, Winter DR, Benjamin AM, Corcoran DL, Ni T (2011). Transcription initiation patterns indicate divergent strategies for gene regulation at the chromatin level.. PLoS Genet.

[37] Mavrich TN, Jiang C, Ioshikhes IP, Li X, Venters BJ (2008). Nucleosome organization in the Drosophila genome.. Nature.

[38] Gilchrist DA, Dos Santos G, Fargo DC, Xie B, Gao Y (2010). Pausing of RNA polymerase II disrupts DNA-specified nucleosome organization to enable precise gene regulation.. Cell.

[39] Muse GW, Gilchrist DA, Nechaev S, Shah R, Parker JS (2007). RNA polymerase is poised for activation across the genome.. Nat Genet.

[40] Juven-Gershon T, Hsu JY, Theisen JW, Kadonaga JT (2008). The RNA polymerase II core promoter - the gateway to transcription.. Curr Opin Cell Biol.

[41] Pugh BF (1996). Mechanisms of transcription complex assembly.. Curr Opin Cell Biol.

[42] FitzGerald PC, Sturgill D, Shyakhtenko A, Oliver B, Vinson C (2006). Comparative genomics of Drosophila and human core promoters.. Genome Biol.

[43] Ohler U, Liao GC, Niemann H, Rubin GM (2002). Computational analysis of core promoters in the Drosophila genome.. Genome Biol.

[44] Thomas-Chollier M, Hufton A, Heinig M, O'Keeffe S, Masri NE (2011). Transcription factor binding predictions using TRAP for the analysis of ChIP-seq data and regulatory SNPs.. Nat Protoc.

[45] Feller C, Prestel M, Hartmann H, Straub T, Soding J (2012). The MOF-containing NSL complex associates globally with housekeeping genes, but activates only a defined subset.. Nucleic Acids Res.

[46] Hochheimer A, Zhou S, Zheng S, Holmes MC, Tjian R (2002). TRF2 associates with DREF and directs promoter-selective gene expression in Drosophila.. Nature.

[47] van Werven FJ, van Teeffelen HA, Holstege FC, Timmers HT (2009). Distinct promoter dynamics of the basal transcription factor TBP across the yeast genome.. Nat Struct Mol Biol.

[48] Mizzen CA, Yang XJ, Kokubo T, Brownell JE, Bannister AJ (1996). The TAF(II)250 subunit of TFIID has histone acetyltransferase activity.. Cell.

[49] Sermwittayawong D, Tan S (2006). SAGA binds TBP via its Spt8 subunit in competition with DNA: implications for TBP recruitment.. EMBO J.

[50] Warfield L, Ranish JA, Hahn S (2004). Positive and negative functions of the SAGA complex mediated through interaction of Spt8 with TBP and the N-terminal domain of TFIIA.. Genes Dev.

[51] Black JC, Choi JE, Lombardo SR, Carey M (2006). A mechanism for coordinating chromatin modification and preinitiation complex assembly.. Mol Cell.

[52] Langmead B, Trapnell C, Pop M, Salzberg SL (2009). Ultrafast and memory-efficient alignment of short DNA sequences to the human genome.. Genome Biol.

[53] Zhang Y, Liu T, Meyer CA, Eeckhoute J, Johnson DS (2008). Model-based analysis of ChIP-Seq (MACS).. Genome Biol.

[54] Salmon-Divon M, Dvinge H, Tammoja K, Bertone P (2010). PeakAnalyzer: genome-wide annotation of chromatin binding and modification loci.. BMC Bioinformatics.

[55] Anders S, Huber W (2010). Differential expression analysis for sequence count data.. Genome Biol.

[56] Conrad T, Cavalli FM, Holz H, Hallacli E, Kind J (2012). The MOF chromobarrel domain controls genome-wide H4K16 acetylation and spreading of the MSL complex.. Dev Cell.

[57] Goecks J, Nekrutenko A, Taylor J (2010). Galaxy: a comprehensive approach for supporting accessible, reproducible, and transparent computational research in the life sciences.. Genome Biol.

[58] Quinlan AR, Hall IM (2010). BEDTools: a flexible suite of utilities for comparing genomic features.. Bioinformatics.

[59] Oliveros JC (2007). VENNY.. An interactive tool for comparing lists with Venn Diagrams.

[60] Vaquerizas JM, Kummerfeld SK, Teichmann SA, Luscombe NM (2009). A census of human transcription factors: function, expression and evolution.. Nat Rev Genet.

[61] Kind J, Vaquerizas JM, Gebhardt P, Gentzel M, Luscombe NM (2008). Genome-wide analysis reveals MOF as a key regulator of dosage compensation and gene expression in Drosophila.. Cell.

[62] Bailey TL, Williams N, Misleh C, Li WW (2006). MEME: discovering and analyzing DNA and protein sequence motifs.. Nucleic Acids Res.

[63] Roider HG, Manke T, O'Keeffe S, Vingron M, Haas SA (2009). PASTAA: identifying transcription factors associated with sets of co-regulated genes.. Bioinformatics.

[64] Bailey TL, Elkan C (1994). Fitting a mixture model by expectation maximization to discover motifs in biopolymers.. Proc Int Conf Intell Syst Mol Biol.

[65] Tomancak P, Berman BP, Beaton A, Weiszmann R, Kwan E (2007). Global analysis of patterns of gene expression during Drosophila embryogenesis.. Genome Biol.

