# Supplementary figures and images for: The NSL Complex Regulates Housekeeping Genes in Drosophila
Source: PLoS Genet. 2012 Jun 14;8(6):e1002736. doi: 10.1371/journal.pgen.1002736 (PMC3375229; doi:10.1371/journal.pgen.1002736)

# Supplementary Figure 1

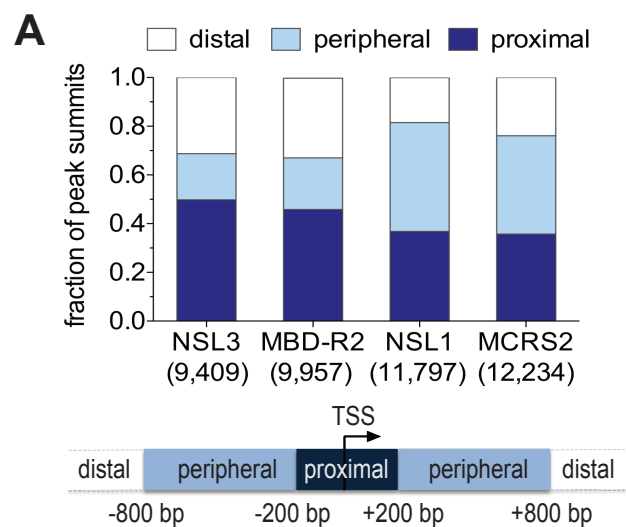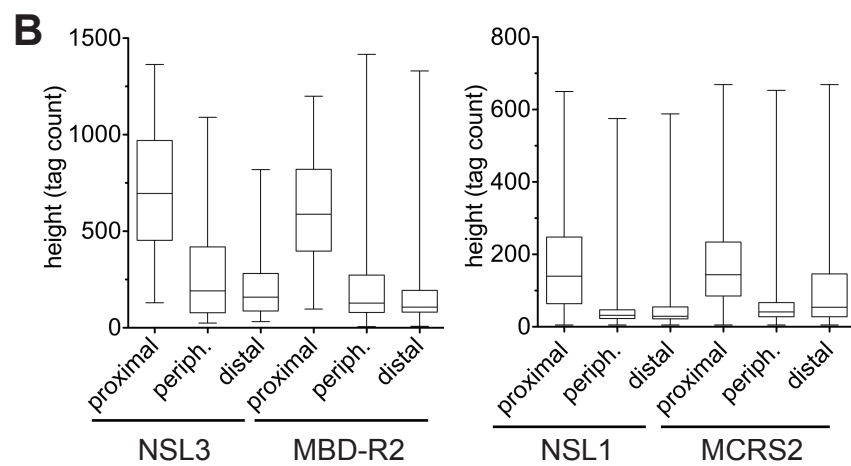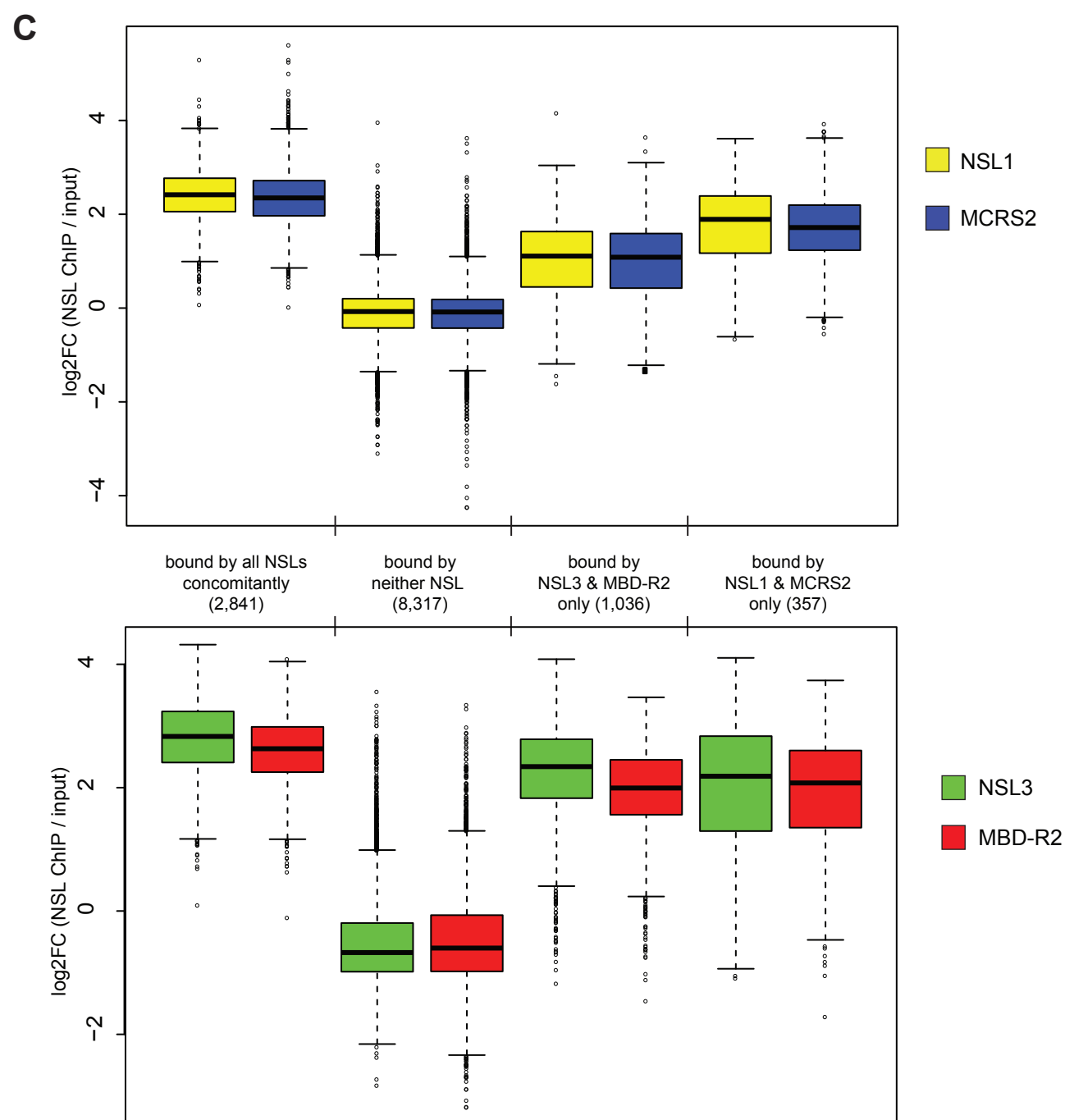

Supplement: Figure S1 — General characteristics of NSL binding profiles. (A) ChIP-Seq peaks obtained from NSL profiles were classified according to their distance from the nearest annotated TSS. The bar chart shows that the majority of NSL binding events is closely associated with annotated TSSs: 68.7% of NSL3 peaks, 67% of MBD-R2 peaks, 81.5% of NSL1 peaks, and 76.1% of MCRS2 peaks localize within 800 bp up- or downstream of the nearest TSS. The schematic diagram below the bar chart visualizes our definitions: proximal peaks localize within +/−200 bp (dark blue), peripheral peaks between 201–800 bp (light blue) and distal peaks are farther away than 800 bp from a TSS (white). (B) The strongest signals of NSL binding are observed within 200 bp of annotated TSSs. This is shown by the box plot of tag counts of peak summits classified as TSS-proximal, -peripheral, or –distal (whiskers = 2.5–97.5 percentiles). (C) The lack of complete overlap of NSL target genes is mainly due to stringent criteria for defining target genes. In Figure 1B, 1,036 genes were shown as “bound by NSL3 and MBD-R2 only” and 357 genes as “bound by NSL1 and MCRS2 only”. We therefore addressed whether these two groups could constitute gene sets that are specific for S2 cells or salivary glands. For this purpose, input-normalized ChIP-seq signals for the promoters for each group of genes were extracted, including those that are bound by all or neither NSL proteins. The box plot shows that the signal of NSL1 and MCRS2 is still significantly higher in those genes that were labeled as “bound by NSL3 and MBD-R2 only” than for those that were defined as NSL-non-bound (p-value<2.2e-16, Wilcoxon test). The same holds true for NSL3 and MBD-R2. Therefore, differences in gene sets are very likely not due to tissue-specific binding, rather to the choice of a very stringent cut-off for the binary decision “bound” or “not-bound”. For details about our definition of NSL target genes, see Materials and Methods and Figure 1B. (PDF) [file pgen.1002736.s001.pdf]

Supplementary Figure 2

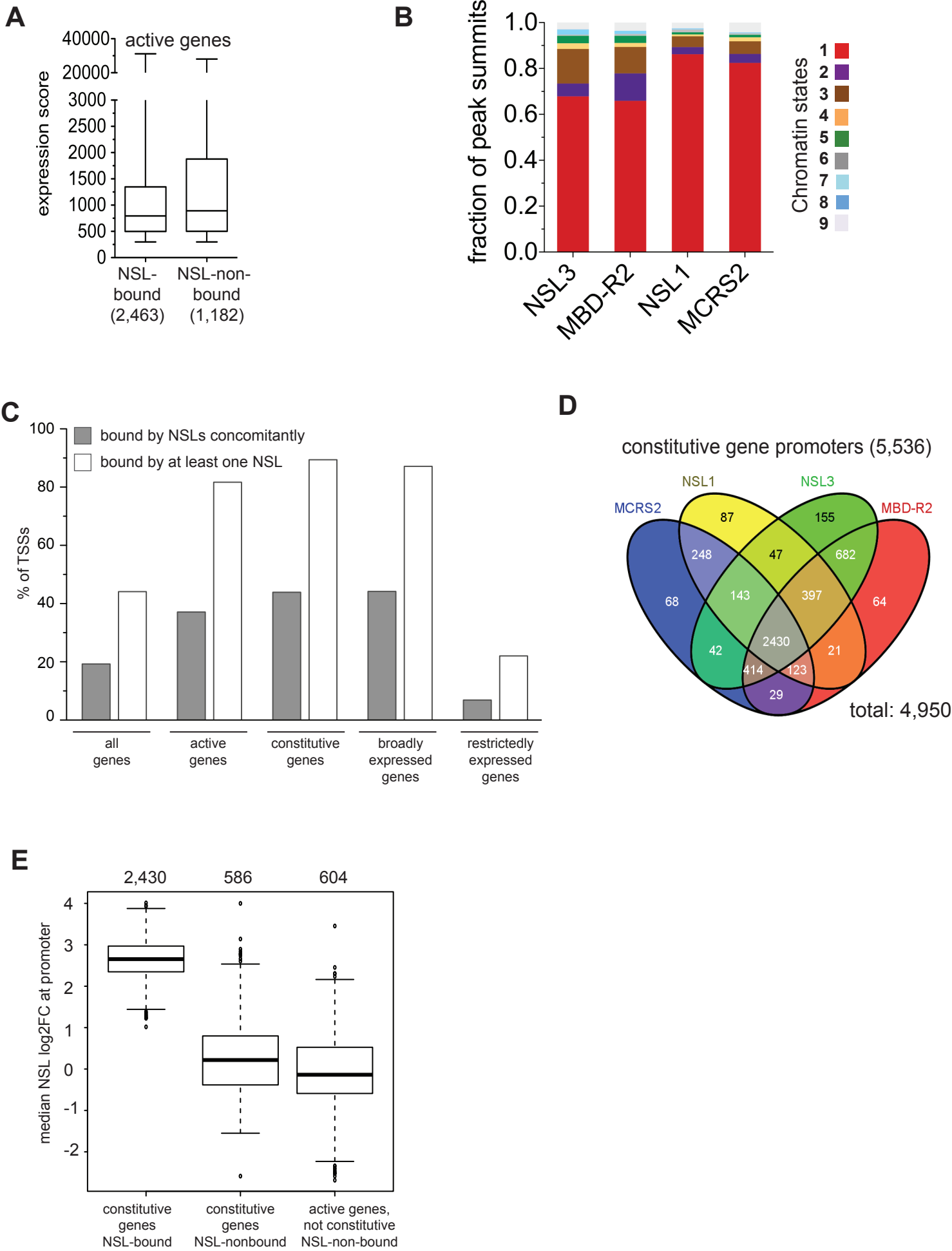

Supplement: Figure S2 — Assessing the overlaps of NSL signals on gene promoters. (A) Median expression levels between expressed genes that are bound by all four NSLs concomitantly do not differ significantly from expressed genes devoid of NSL binding as shown by the box plot (whisker = 2.5–97.5 percentiles). The expression scores were taken from [28]. (B) The NSL complex preferentially binds to regions of open and actively transcribed chromatin (state 1, [30]) as peak summits intersected with the regions reported by [30] are dramatically enriched for state 1 (regardless of their localization). (C) Overview of TSS-associated NSL binding: 19.25% of annotated TSSs are bound by NSL1, MCRS2, NSL3, and MBD-R2 concomitantly. When looking at the subsets of active and housekeeping genes, the numbers increase to 37.1% (active) and 43.9% (constitutive) that are bound by all four NSLs across different cell types and experiments. To confirm the findings that were based on our own definition of housekeeping genes (see Materials and Methods), we also tested a previously published set of broadly and restrictedly expressed genes [65]. (D) The Venn diagram shows the individual overlaps of the gene promoters bound by the single NSL proteins. The core intersect (2,430) corresponds to the gray bar of “constitutive genes” in Figure S2C, while the total number of 4,950 represents the number of constitutive TSSs bound by at least one NSL. (E) Constitutive genes classified as NSL-non-bound according to our criteria described in Materials and Methods (see Figure 1 for visualization) show slightly, but significantly elevated levels of NSL binding compared to non-constitutively expressed genes. This verifies the preference of the NSL complex for housekeeping genes and suggests that some constitutive genes classified as NSL-non-bound were missed due to the cut-off we used for all four samples. The boxplot shows the median log2FCs (ChIP/input) for the 400 bp regions centered around TSSs. The medians were calculated for [file pgen.1002736.s002.pdf]

Supplementary Figure 3

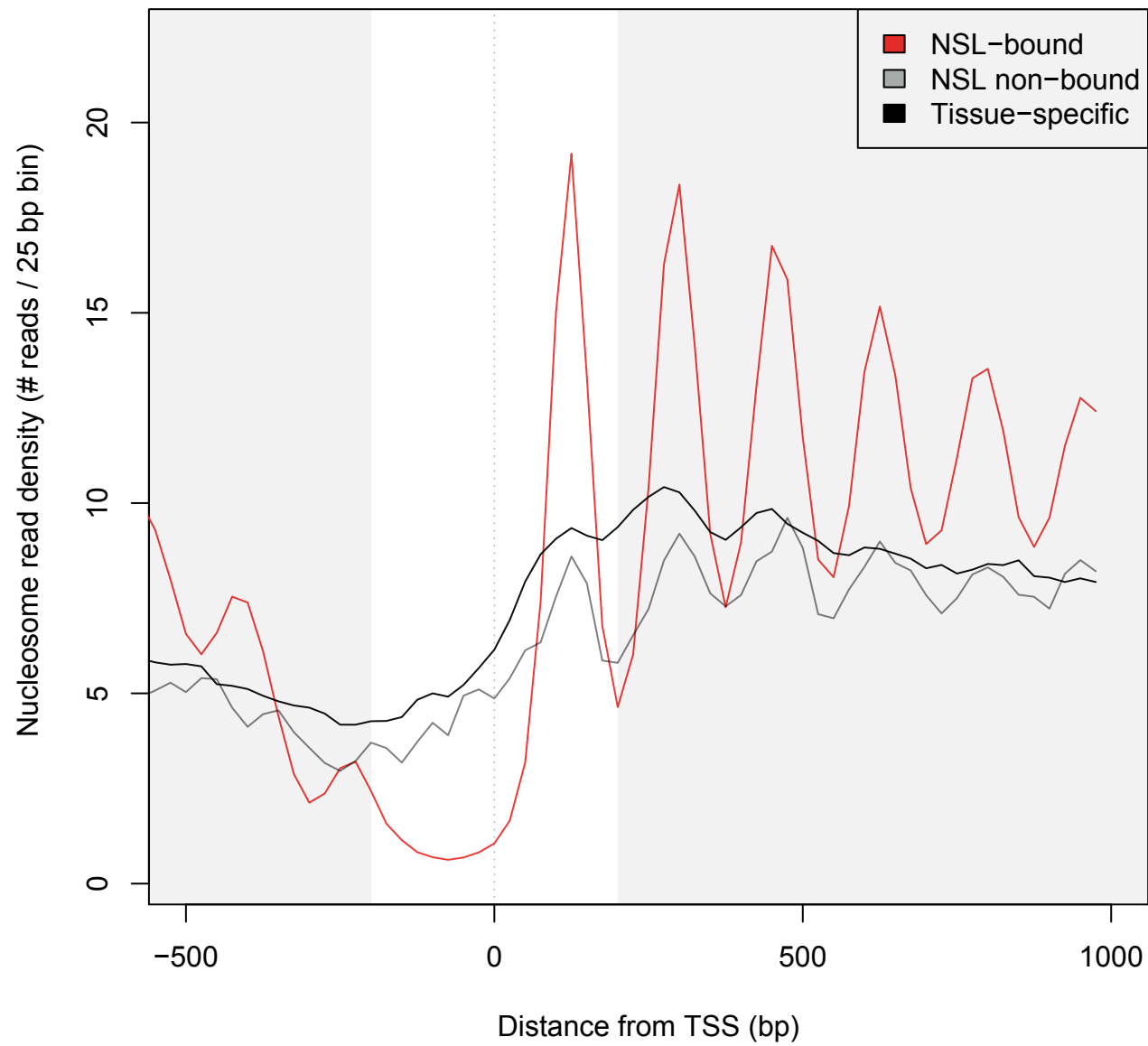

Supplement: Figure S3 — NSL-bound and NSL-non-bound housekeeping genes display different nucleosome organizations. Nucleosome occupancy metaprofiles for NSL-bound (red), constitutively expressed NSL-non-bound (gray) and tissue-specific (black) genes. Metaprofiles were calculated for each group as the sum of nucleosome reads overlapping 25 bp bins spanning the −500/+1000 bp region centered at the TSS of each gene. The non-shaded white area corresponds to the −200/+200 bp region used for the analysis in Figure 3B. (PDF) [file pgen.1002736.s003.pdf]

## Supplementary Figure 4

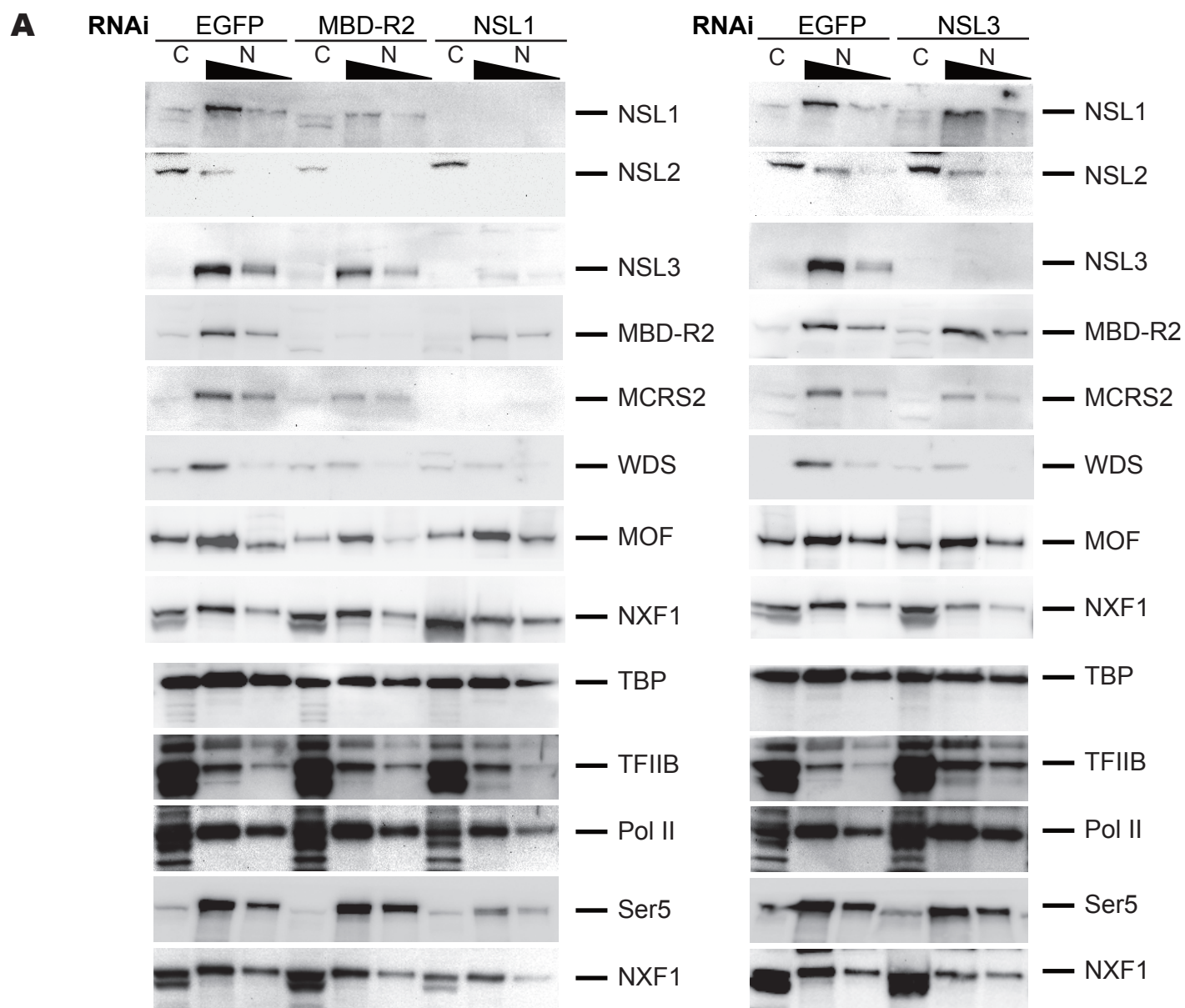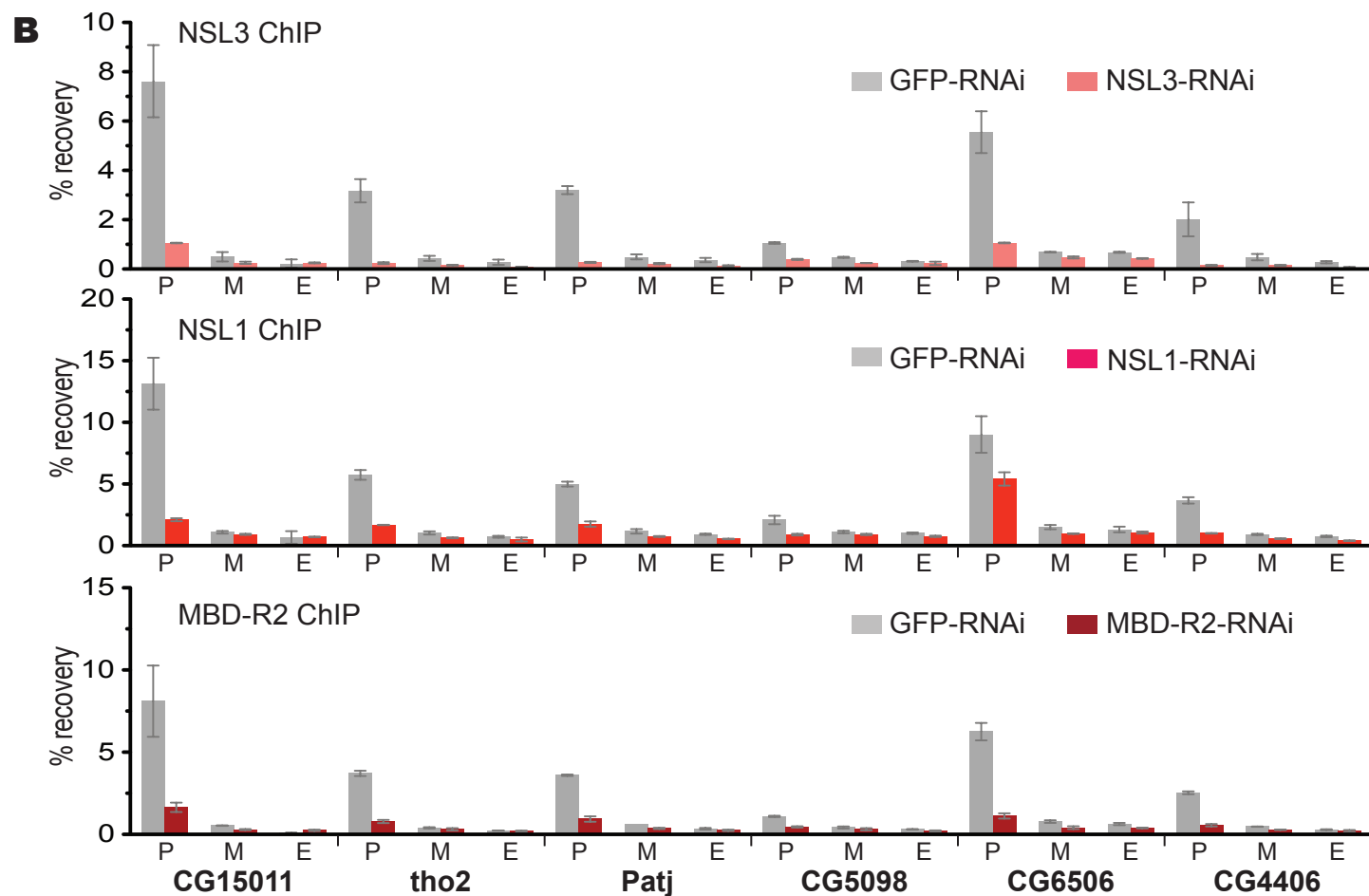

Supplement: Figure S4 — Depletion of different NSL proteins have distinct effects on the stability of the remaining NSL complex members but not for Pol II machinery components. (A) Western blot analyses of cytoplasmic (C) and nuclear (N) extracts from S2 cells that had been treated with dsRNA against GFP, MBD-R2, NSL1, and NSL3. Depletion of NSL1 greatly affects the stability of other NSL complex proteins namely: NSL2, NSL3, MCRS2, MBD-R2 and WDS. Depletion of NSL3 or MBD-R2 has milder effects on the levels of other NSL proteins. MOF protein levels appear affected upon MBD-R2 depletion but not in NSL1 or NSL3 knockdowns. In contrast, TBP, TFIIB and Pol II are only modestly affected in either knockdown especially when taking into consideration the loading control Nuclear RNA export factor 1 (NXF1). (B) To check whether the dsRNA treatment against NSL3, NSL1, and MBD-R2 efficiently reduced NSL binding to its target regions, ChIP was performed with antibodies against NSL1, NSL3 and MBD-R2 in the respective knockdowns in S2 cells. GFP-RNAi was used as a control. “P”, “M”, “E” represent promoter, middle and end of gene, respectively. Error bars represent the standard deviation of three independent experiments. (PDF) [file pgen.1002736.s004.pdf]

# Supplementary Figure 5

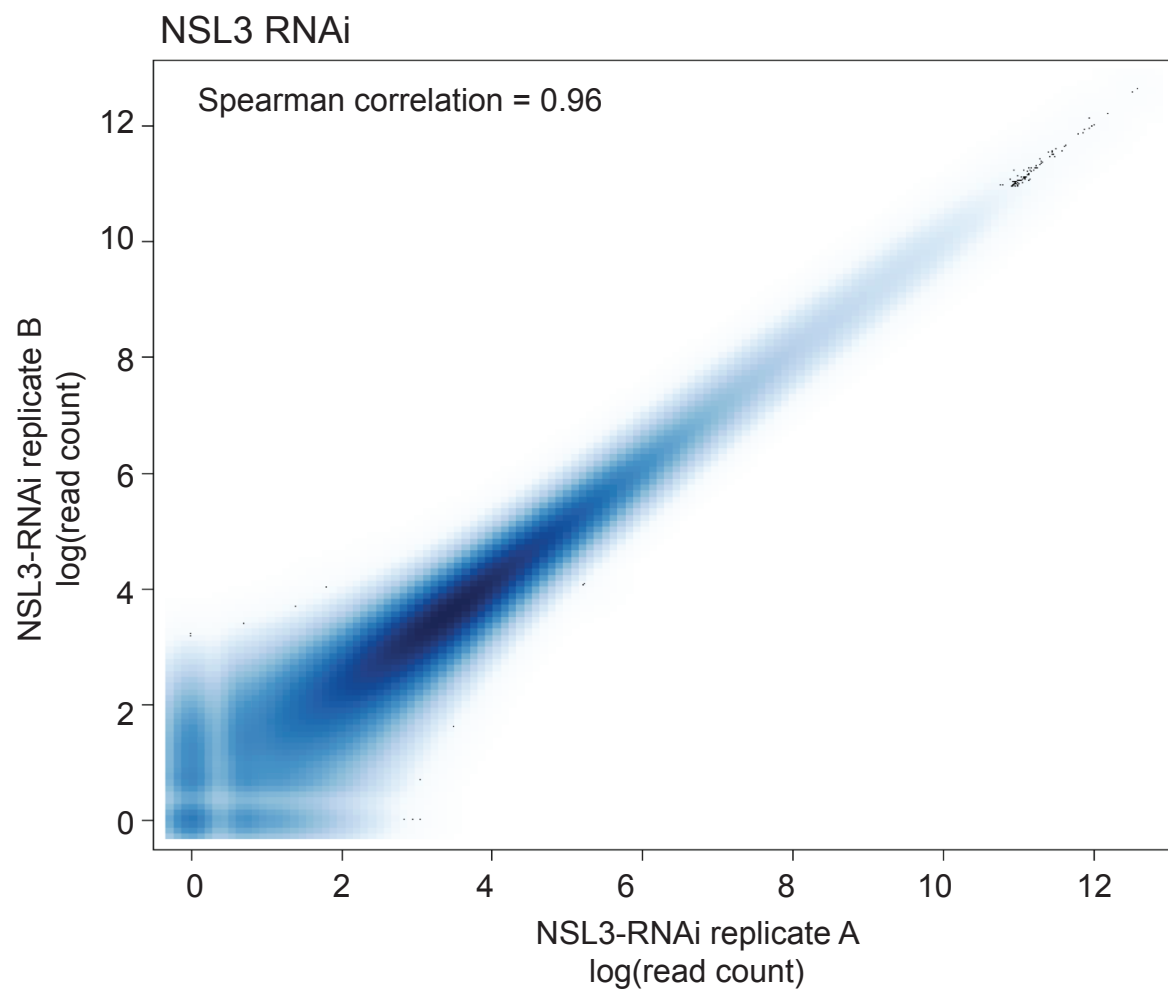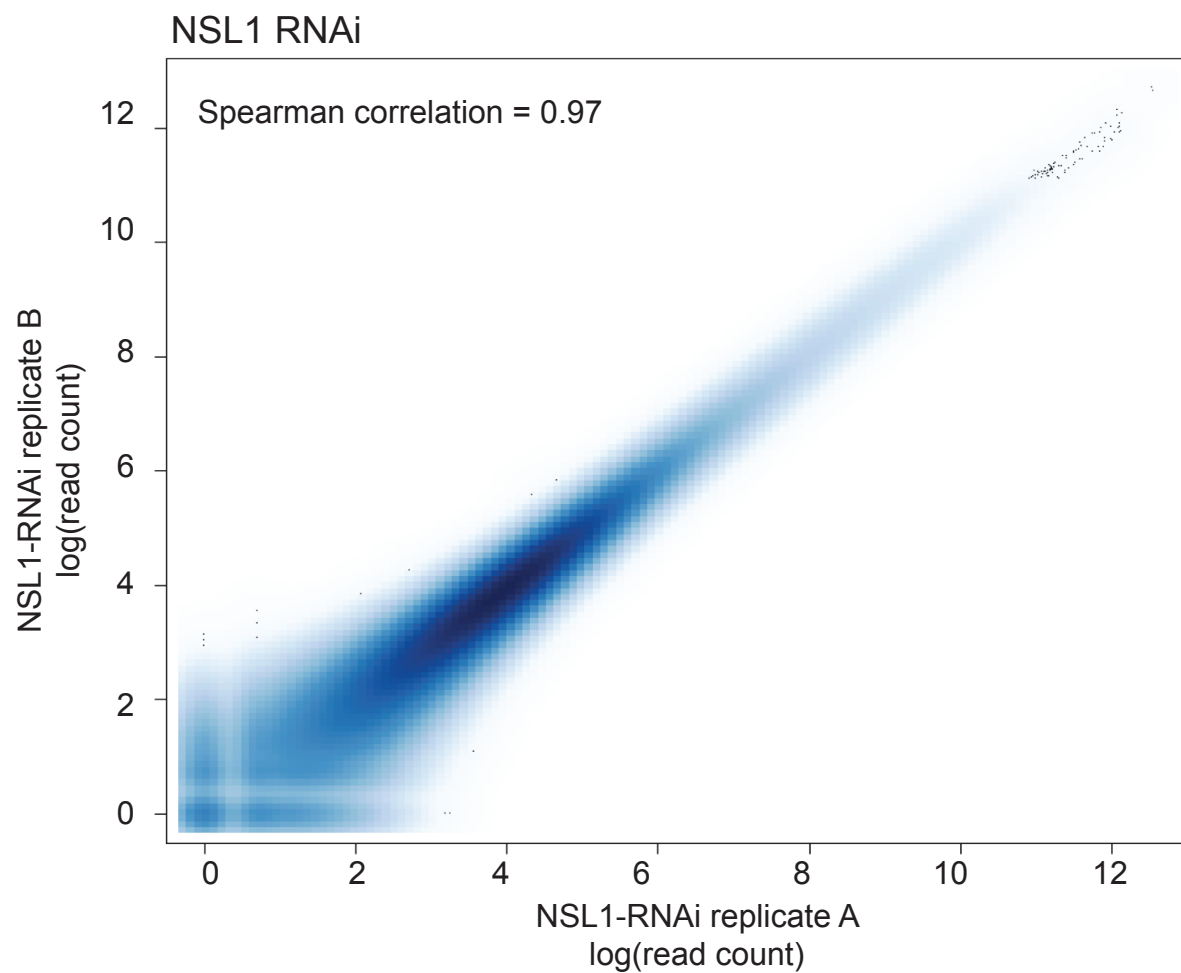

Supplement: Figure S5 — Correlation of biological duplicates for the ChIP-seq of Pol II in knockdowns of NSL1 and NSL3. Correlation plots between the two Pol II ChIP-seq libraries generated from duplicate knockdown experiments for (a) NSL3 and (b) NSL1. Reads were mapped to the genome with bowtie. The read counts plotted here were extracted for 25 bp bins along the entire D. melanogaster genome. The Spearman correlations for the biological replicates are excellent (0.96 for NSL3-RNAi samples, 0.97 for NSL1-RNAi samples). (PDF) [file pgen.1002736.s005.pdf]

Supplementary Figure 6

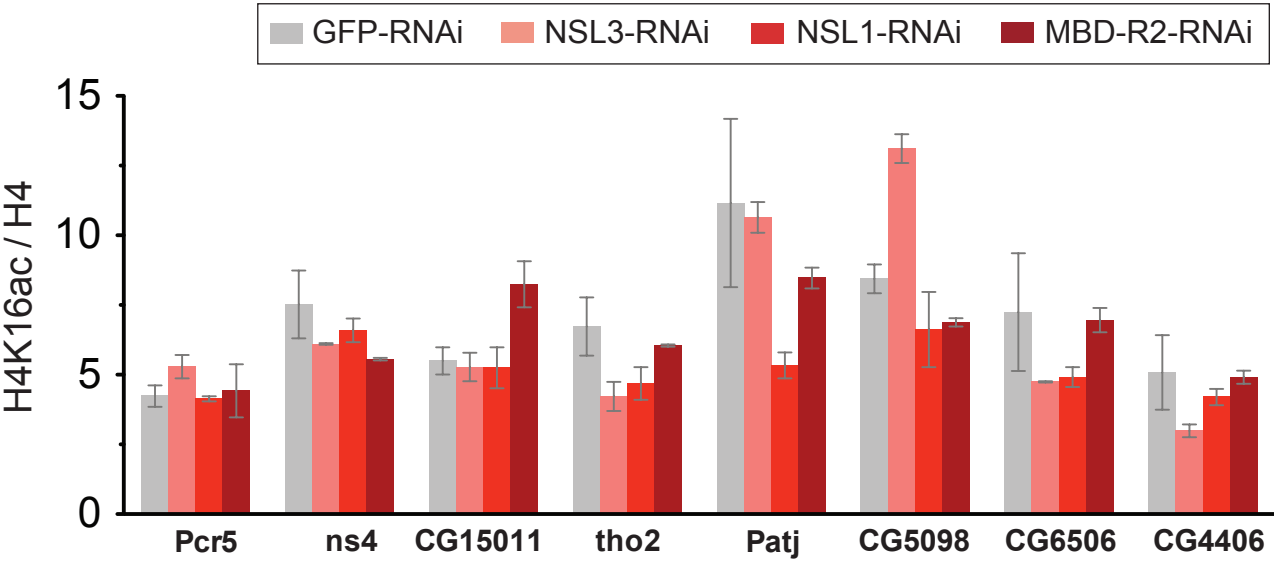

Supplement: Figure S6 — Chromatin immunoprecipitation of H4K16ac in NSL1, NSL3 and MBDR2 depleted cells. ChIP-qPCR was performed using antibodies against H4K16ac and H4 in NSL1, NSL3 or MBD-R2 depleted cells. The H4K16ac signal is normalized against H4 signal from the same region. Consistent with our previous results, H4K16ac is very modestly reduced upon depletion of NSL complex members. The quantitative qPCR was performed on 5 autosomal genes (P5cr, ns4, CG15011, tho2, Patj, CG5098) as well as 2 X-linked genes (CG6506 and CG4406). Primers were positioned at the promoter of the indicated genes. Error bars represent the standard deviation of three independent experiments. (PDF) [file pgen.1002736.s006.pdf]

Supplementary Figure 8

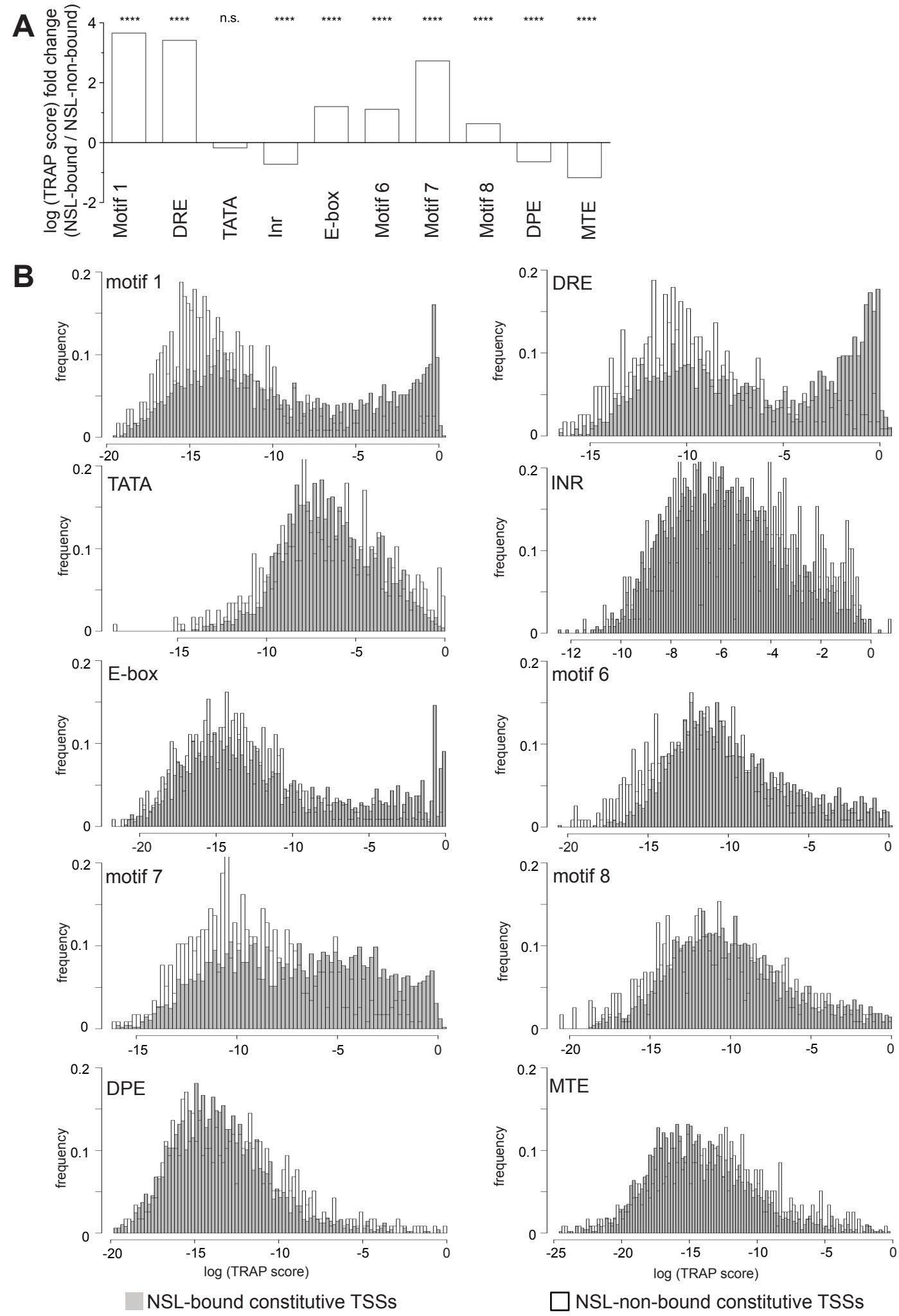

Supplement: Figure S8 — Comparison of motif enrichments for NSL-bound and –non-bound constitutively active TSSs. (A) The bar chart displays the fold change of the core promoter affinities for the sequences of NSL-bound promoters (concomitant binding of NSL1, MCRS2, MBD-R2, NSL3) compared to NSL-non-bound promoters (not bound by any of the NSL proteins). The bar chart shows that even when motif enrichments are calculated within the subset of constitutively active genes, NSL-bound promoters are enriched for motif 1, DRE, E-box, motif 6, 7 and 8 whereas the depletion of TATA box, Inr motif, DPE and MTE becomes less evident. P-values were calculated with two-sided Wilcoxon rank sum test, **** = P<0.0001, *** = P<0.001, ** = P<0.01, * = P<0.5, not significant (n.s.) = P>0.5. The fold change was calculated as log(median(TRAP score of NSL-bound promoters)/median(TRAP score of NSL-non-bound promoters)). (B) Individual TRAP score [44], [63] histograms for the 10 core promoter motifs [43] that underlie the bar chart of S6A. The histograms show the distributions of the motif affinities for NSL-bound and –non-bound promoters of housekeeping genes. The visible shifts towards higher or lower TRAP scores in NSL-bound or –non-bound genes, respectively, represent the fold changes seen in the bar chart. (PDF) [file pgen.1002736.s008.pdf]

Supplementary Figure 9

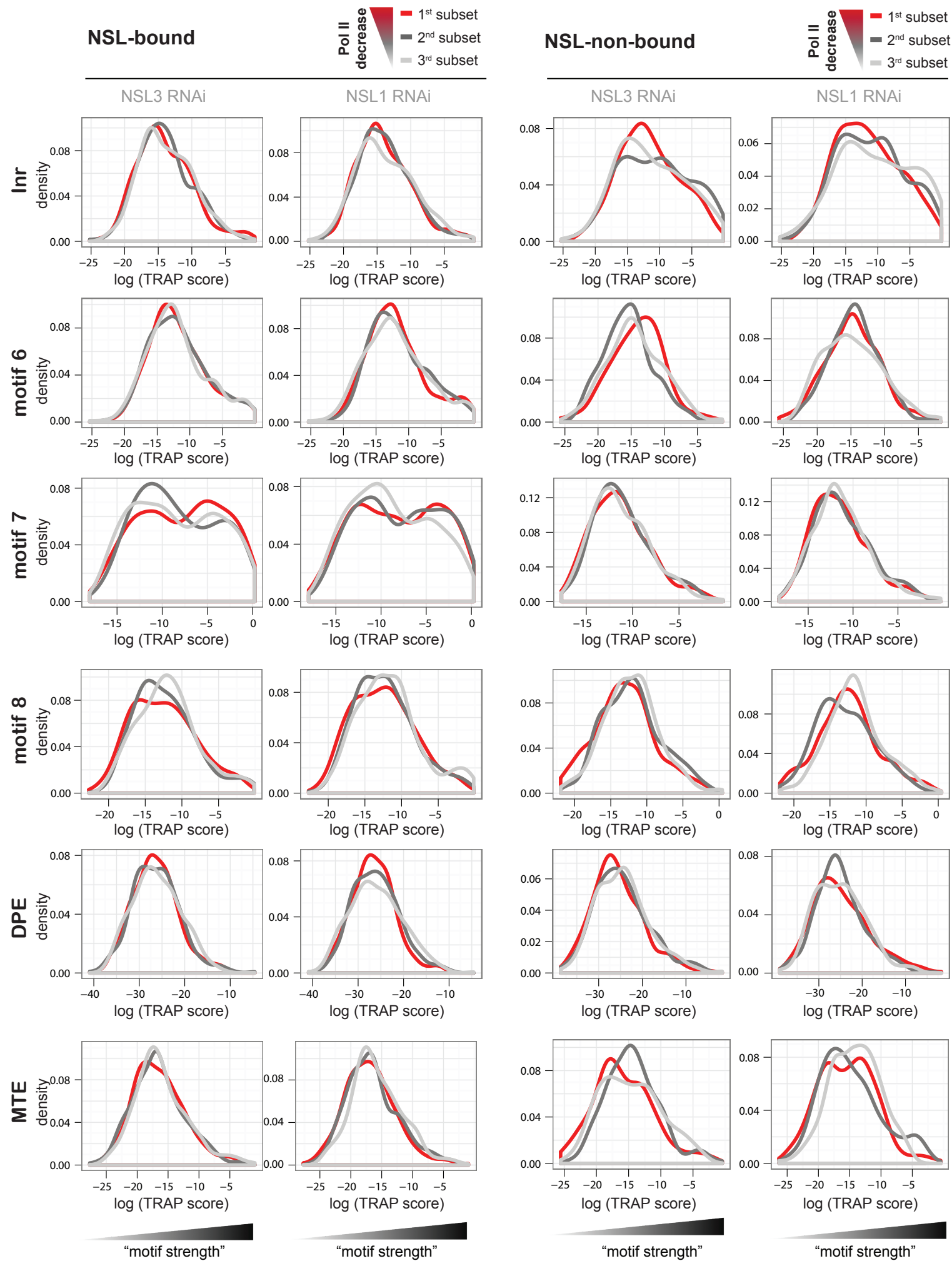

Supplement: Figure S9 — TRAP score densities of NSL-bound and –non-bound genes. We selected non-overlapping genes that showed significant Pol II binding in control samples and reduced Pol II levels in NSL knockdown conditions and sorted them into three groups according to the magnitude of Pol II loss. The 1st subset (red line) contains genes with the strongest reduction of Pol II in promoter regions; the 3rd subset (gray line) correspondingly contains genes with smallest Pol II loss. TRAP score was calculated as a measure of protein binding affinity towards the known promoter motifs identified by Ohler et al. [43]. Of the motifs shown here, only motif 7 displays a moderate association between the motif's strength and Pol II loss upon NSL depletion (for remaining motifs see Figure 6C). (PDF) [file pgen.1002736.s009.pdf]
