# Supplementary material for: The NSL Complex Regulates Housekeeping Genes in Drosophila
Source: PLoS Genet. 2012 Jun 14;8(6):e1002736. doi: 10.1371/journal.pgen.1002736 (PMC3375229; doi:10.1371/journal.pgen.1002736)
Supplement: Figure S7 — De novo motif identification in NSL binding regions. (A) Motifs identified by MEME in peak summit regions of NSL3, MBD-R2, NSL1, and MCRS2. Results of MEME analyses of peaks ranked 501–1,000 (r.c. = reverse complement) confirm the motifs identified in the 500 highest peaks as shown in Figure 6A. (B) Results of MEME analyses of 500 peak summits that were not selected solely according to their height, but also on the basis of their association with constitutively expressed genes. The motifs and their occurrences recapitulate the results from the analysis of the highest intensity peaks (Figure 6A), reinforcing the preference of NSL targeting to genomic regions containing the motifs shown above. (PDF) [file pgen.1002736.s007.pdf]

Supplementary Figure 7

A Peak summits with tag count ranks 500 - 1,000

Motif	Logo		NSL3	MBD-R2	NSL1	MCRS2
DRE	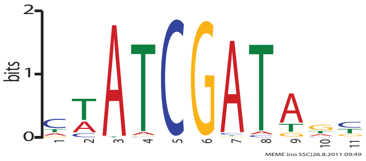	sites<br>E-value	—	—	183/500<br>6.5×10^{-188}	123/500<br>3.4×10^{-104}
E-box	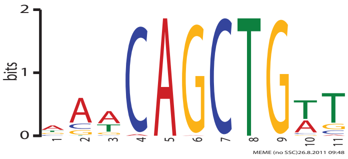	sites<br>E-value	117/500<br>3.4×10^{-115}	83/500<br>1.3×10^{-54}	77/500<br>1.7×10^{-61}	90/500<br>8.3×10^{-91}
DMv2-like (r.c.)	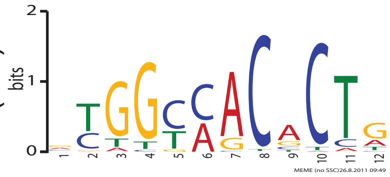	sites<br>E-value	—	—	263/500<br>3.9×10^{-189}	222/500<br>6.4×10^{-146}
Motif 1 (r.c.) (Ohler)	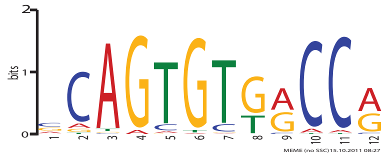	sites<br>E-value	64/500<br>4.2×10^{-35}	63/500<br>1.7×10^{-42}	—	—

B Peak summits of peaks associated with constitutive genes

Motif	Logo		NSL3	MBD-R2	NSL1	MCRS2
DRE	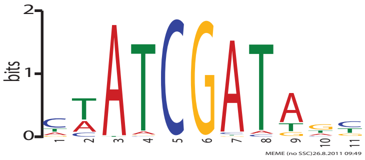	sites<br>E-value	—	—	229/500<br>3.3×10^{-215}	184/500<br>1.9×10^{-121}
E-box	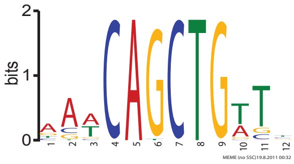	sites<br>E-value	152/500<br>2.3×10^{-234}	103/500<br>8.6×10^{-109}	59/500<br>2.3×10^{-31}	101/500<br>1.7×10^{-80}
DMv2-like (r.c.)	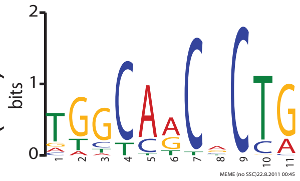	sites<br>E-value	—	295/500<br>1.3×10^{-25}	295/500<br>2.5×10^{-295}	337/500<br>2.6×10^{-276}
Motif 1 (r.c.) (Ohler)	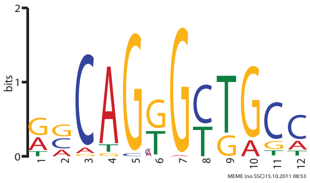	sites<br>E-value	62/500<br>4.3×10^{-33}	—	—	—
