# Supplementary material for: The NSL Complex Regulates Housekeeping Genes in Drosophila
Source: PLoS Genet. 2012 Jun 14;8(6):e1002736. doi: 10.1371/journal.pgen.1002736 (PMC3375229; doi:10.1371/journal.pgen.1002736)
Supplement: Table S1 — Overview of the numbers of NSL peaks overlapping with annotated TSSs. The numbers of peaks (regions of significant NSL binding signals) determined by MACS [53] are comparable between the different proteins. However, the increased sequencing depth of the NSL3 and MBD-R2 ChIP-seq experiments led the detection of expanded regions of NSL binding signals that is reflected by more widespread peak. The PeakSplitter algorithm [54] divides peaks identified by MACS at sites of local maxima. (PDF) [file pgen.1002736.s010.pdf]

Table S1

		NSL1	MCRS2	NSL3	MBD-R2
peaks (MACS)		3,541	3,733	4,244	3,399
median peak size		1,129 bp	1,314 bp	2,227 bp	2,676 bp
(sub)peaks (PeakSplitter)		11,797	12,234	9,409	9,957
subpeaks per peak (median)		3	3	2	2
median peak size		350 bp	379 bp	1,110 bp	1,040 bp
TSSs bound	general	28.7 %	28.9 %	36.8 %	36.1 %
	active	55.7 %	54.1 %	68.3 %	65.9 %
	constitutive	63.2 %	63.2 %	77.9 %	75.2 %
